# The cuproptosis related genes signature predicts the prognosis and correlates with the immune status of clear cell renal cell carcinoma

**DOI:** 10.3389/fgene.2022.1061382

**Published:** 2022-12-01

**Authors:** Peng Sun, Hua Xu, Ke Zhu, Min Li, Rui Han, Jiran Shen, Xingyuan Xia, Xiaojuan Chen, Guanghe Fei, Sijing Zhou, Ran Wang

**Affiliations:** ^1^ Department of Respiratory and Critical Care Medicine, The First Affiliated Hospital of Anhui Medical University, Hefei, China; ^2^ Department of Nursing, Hefei Second People’s Hospital, Hefei, China; ^3^ Department of Oncology, The First Affiliated Hospital of Anhui Medical University, Hefei, China; ^4^ Department of Infectious Diseases, Hefei Second People’s Hospital, Hefei, China; ^5^ Department of Occupational Disease, Hefei Third Clinical College of Anhui Medical University, Hefei, China

**Keywords:** cuproptosis, clear cell renal cell carcinoma, immune, ceRNAs network, TCGA

## Abstract

**Background:** Clear cell renal cell carcinoma (CCRCC) has a high incidence and poor prognosis. Cuproptosis, an independent pattern of cell death associated with copper, plays an important role in cancer proliferation and metastasis. The role of cuproptosis-related genes (CRGs) in CCRCC is unclear.

**Methods:** Transcriptome and clinical information for CCRCC were downloaded from The Cancer Genome Atlas (TCGA) database. After dividing the training and testing cohort, a 4-CRGs risk signature (*FDX1*, *DLD*, *DLAT*, *CDKN2A*) was identified in the training cohort using Least absolute shrinkage and selection operator (LASSO) and Cox regression analysis. The effect of the 4-CRGs risk signature on prognosis was assessed using Kaplan-Meier (KM) curves and time-dependent receiver operating characteristic (ROC) curves and verified using the testing cohort. For different risk groups, the immune statue was assessed using the CIBERSORT algorithm, the ssGSEA method and immune checkpoint expression data. Finally, a competitive endogenous RNA (ceRNA) network was constructed using miRTarbase and starBase databases to identify molecules that may have a regulatory relationship with CRCCC.

**Results:** There were significant changes in the overall survival (OS), immune microenvironment, immune function, and checkpoint gene expression among the different risk groups. A ceRNA network consisting of one mRNA, two miRNAs, and 12 lncRNAs was constructed.

**Conclusion:** The 4-CRGs risk signature provides a new method to predict the prognosis of patients with CCRCC and the effect of immunotherapy. We propose a new cuproptosis-associated ceRNA network that can help to further explore the molecular mechanisms of CCRCC.

## 1 Introduction

Renal cell carcinoma (RCC) is one of the top ten most common cancers in the world, ranking sixth and eighth in new cases in men and women, respectively (Siegel et al., 2020). Clear cell renal cell carcinoma (CCRCC), is the most common type of RCC, accounting for about 75–80% of RCCs (Nabi et al., 2018). Early diagnosis of RCC is difficult because only 6–10% of patients present with typical symptoms, such as hematuria, back pain, or abdominal mass (Patard et al., 2003). Furthermore, the effect of chemotherapy and radiation therapy in patients with CCRCC is not ideal, and tumor removal is the best treatment option (Sonpavde et al., 2012). Immunotherapy is an emerging and promising therapeutic option, and some immune checkpoint inhibitors (ICIs) have been approved for metastatic CCRCC (nivolumab) after failed targeted therapy or in combination with targeted drugs (pembrolizumab + axitinib/avelumab + axitinib) as first-line therapy (Motzer et al., 2015; Motzer et al., 2019; Rini et al., 2019). However, in actual clinical practice, there are still problems regarding which treatment methods can be used for individual patients, especially advanced patients. Therefore, it is necessary to establish a reliable predictive model to predict patient survival and guide the choice of different treatment options. We aimed to identify potential targets with prognostic implications for CCRCC from the perspective of cuproptosis, a newly discovered pattern of cell death.

Copper accumulates within cells and can induce cell death when a certain concentration is reached (Tsvetkov et al., 2022). In tumors, copper is involved in cell proliferation, epithelial-mesenchymal transition (EMT), angiogenesis, immunity, inflammation, and metastasis of tumors (De Luca et al., 2019; da Silva et al., 2022). Copper chelation may inhibit these processes to exert anti-tumor and anti-metastatic effects (Denoyer et al., 2015; Shanbhag et al., 2021). Copper ionophores have recently been shown to induce a novel mechanism of cell death (cuproptosis) (Tsvetkov et al., 2022). There have been studies on copper ion carriers that play a role in anti-tumor activity. For example, disulfiram has a significant tumor growth inhibition effect in patients with prostate (Safi et al., 2014) and breast cancer (Zhang et al., 2010; Allensworth et al., 2015). Disulfiram can improve the survival rate of patients treated with cisplatin and vinorelbine for non-small cell lung cancer (Nechushtan et al., 2015). Cuproptosis-related genes may serve as new targets for cancer treatment, but there are few studies on copper ionophores and cuproptosis.

Here, we downloaded the TCGA-KIRC dataset to identify differential genes associated with cuproptosis in tumor tissue and normal samples and validated them using the GSE53757 dataset. A risk score model containing four cuproptosis-related genes was constructed using LASSO and Cox regression, and the correlation between the risk score model and immune function, immune infiltrates, immune escape, and cancer treatment drugs were analyzed. Finally, a possible ceRNA network was constructed by searching for miRNAs and lncRNAs associated with cuproptosis-related genes using TCGA, miRTarbase, and starBase databases.

## 2 Materials and methods

### 2.1 Data collection

The RNA-sequencing TPM data and corresponding clinical data of KIRC were retrieved from the TCGA database (https://portal.gdc.cancer.gov/), including 541 KIRC samples with complete survival data and 72 normal samples. The RNA-sequencing TPM data and corresponding clinical data of LGG were retrieved from the TCGA database (https://portal.gdc.cancer.gov/), including 479 LGG samples. The GSE22541 dataset was downloaded from GEO (http://www.ncbi.nlm.nih.gov/geo) and included 68 CCRCC samples with survival data. The GSE53757 dataset was downloaded from GEO (http://www.ncbi.nlm.nih.gov/geo) and included 72 CCRCC samples and 72 normal samples (von Roemeling et al., 2014). *FDX1*, *LIPT1*, *LIAS*, *DLD*, *DLAT*, *PDHA1*, *PDHB*, *MTF1*, *GLS*, and *CDKN2A* are thought to be cuproptosis-related genes and are involved in two structurally distinct copper-loaded ionophores (Tsvetkov et al., 2022).

### 2.2 Expression patterns of cuproptosis-related genes in clear cell renal cell carcinoma

RNA-sequencing TPM data from the TCGA database were used to compare the expression of cuproptosis-related genes in CCRCC specimens and normal specimens using the Wilcoxon rank-sum test. Statistical significance was set at *p* < 0.05. The GSE53757 dataset was used for further validation.

### 2.3 Correlation analysis and GO and KEGG analysis

For significant prognosis-related cuproptosis-related genes, we performed gene co-expression analysis in TCGA CCRCC patients and set the absolute value of the correlation coefficient to greater than 0.4 with a *p*-value less than 0.001 to obtain the co-expression genes. To further understand the potential role of cuproptosis-related genes in CCRCC, GO and KEGG analyses were performed on co-expressed copper death-related genes.

### 2.4 Construction and validation of the 4-CRGs risk signature

We divided the training and testing cohorts into a ratio of 7:3 for patients with CCRCC. Clinical statistical analysis of the training and testing groups was performed using the chi-square test. In the training cohort, a univariate Cox regression analysis of cuproptosis-related genes was performed to identify the significant prognostically related genes. For significant prognosis-related cuproptosis-related genes, we used LASSO regression analysis to obtain independent prognostic genes in the training set. LASSO regression improves the accuracy and interpretability of the model and reduces the risk of overfitting (Tibshirani, 1997). Multivariate Cox regression analysis was conducted to obtain regression coefficients for independent prognostic genes. Finally, a 4-CRGs risk signature was established based on the multivariate Cox regression coefficient beta value, and the formula was as follows: risk score = EXPgene1∗ β1 + EXPgene2∗β2 + EXPgene3∗β3 + . . . + EXPgenen∗βn, where EXP is the expression level and β represents the regression coefficient from the multivariate Cox. In both cohort, by calculating the risk score for each sample, patients were divided into low- and high-risk groups using the median cut-off value. Furthermore, the KM curve was used to compare the overall survival (OS) between the two groups using the log-rank test. A time-dependent ROC curve analysis was used to assess the predictive power of the 4-CRGs risk signature. Finally, we perform external validation with the external validation cohort GSE22451 and TCGA-LGG. In each independent external validation cohort, based on the risk score, patients were classified into two groups. Maximally selected rank statistics was applied by using an R package “survival”, and “survminer” to identify the optimal cutting point to divide patients.

### 2.5 Construction of nomogram

We screened for prognostic predictive factors including clinical characteristics and risk scores. Specifically, the univariate Cox proportional hazard model was employed to analyze the correlation between the risk score and OS, and multivariate Cox regression analysis was used to evaluate whether the established risk score could serve as an independent prognostic predictor. Further, to comprehensively assess patient survival, we constructed a nomogram integrating distinct clinicopathological information, including age, stage, and risk score, using the “rms” package. Additionally, the decision curve analysis (DCA) of 1, 3, and 5 years was calculated to evaluate whether the synthetic nomogram we established was suitable for clinical application.

### 2.6 Immune function, immune infiltrates, immunomodulatory, and drugs

We used the CIBERSORT algorithm to assess the degree of infiltration of 22 immune cells in different CCRCC samples (Newman et al., 2015). Single-sample gene set enrichment analysis (ssGSEA) was applied to explore the different infiltration degrees of immune-related functions in different CCRCC samples of the TCGA database using the R package “GSVA”.

TIMER is a website that can systematically analyze immune infiltration in various malignancies (https://timer.cistrome.org/) (Li et al., 2020). We investigated the relationship between gene expression and gene markers of TILs in CCRCC.

TISIDB (http://cis.hku.hk/TISIDB/index.php) was used to investigate the association of genes with immunostimulators in CCRCC.

The “pRRophetic” R package was used to predict the half-maximal inhibitory concentration (IC50) of some drugs in each sample regarding tumor treatment.

### 2.7 ceRNA network

We used RNAseq data from the TCGA database and miRNAseq data, including 541 KIRC samples and 72 normal samples. The difference analysis was performed using the DESeq2 package, and |logFC|>1 and adj. *p* < 0.05 were set as thresholds to obtain the differential expression of lncRNAs (DElncRNAs) and miRNAs (DEmiRNAs) between CCRCC patients and normal patients. Subsequently, cuproptose-related miRNAs (CRMs) were predicted using the miRTarBase database (Huang et al., 2020). DEmiRNAs and CRMs were intersected to obtain cuproptosis-related DEmiRNAs (CRDEMs). Cuproptosis-related lncRNAs (CRLs) were predicted using the starBase database (Li et al., 2014). CRLs and DElncRNAs were intersected to obtain the cuproptosis-related DElncRNAs (CRDELs). Subsequently, we integrated the interactions between CRDEMs, CRDELs, and cuproptosis-related genes to construct a ceRNA regulatory network. Finally, Cytoscape (version 3.8.0) software was used to visualize the ceRNA regulatory network.

### 2.8 Statistical analysis

All statistical analyses were conducted using R version 4.1.2 software (https://www.r-project.org/). Univariate Cox hazard regression analyses were performed to identify the independent prognostic cuproptosis-related genes. Survival analysis was conducted by the Kaplan-Meier (K-M) method with the log-rank test. We also compared the expression of cuproptosis-related genes at different clinical stages by using the Wilcoxon rank-sum test.

## 3 Results

### 3.1 Identification of differentially expressed cuproptosis-related genes in normal and tumor samples

**FIGURE 1:**
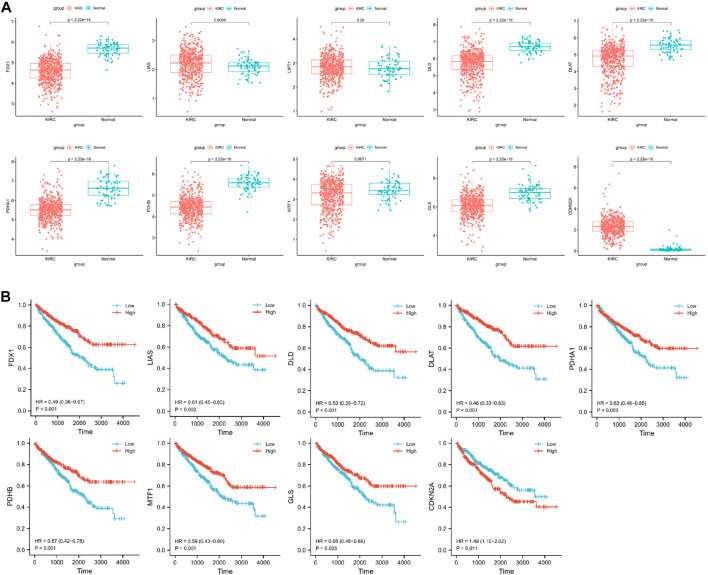
**(A)** Identification of differentially cuproptosis-related expressed genes in TCGA cohort. **(B)** Identification of cuproptosis-related genes with prognostic value.

We compared the expression of cuproptosis-related genes between 541 CCRCC samples and 72 normal samples using the Wilcoxon rank-sum test in the TCGA cohort. We found that *LIAS* and *CDKN2A* were significantly upregulated in tumor samples, and *FDX1*, *DLD*, *DLAT*, *PDHA1*, *PDHB*, *MTF1*, and *GLS* were significantly downregulated in tumor samples (Figure 1A).

### 3.2 Identification of cuproptosis-related genes with prognostic value

To obtain reliable survival results for CCRCC, we first excluded samples with a survival time of less than 30 days. In total, 518 samples were obtained (Table 1). Nine differentially expressed cuproptosis-related genes (*FDX1*, *DLD*, *DLAT*, *PDHA1*, *PDHB*, *MTF1*, *GLS*, *LIAS*, and *CDKN2A*) were identified. Through the KM curve, we found that 9 selected genes all had an impact on the prognosis of CCRCC, including *FDX1* (hazard ratio, HR = 0.49; 95% confidence interval, 95%CI = 0.36–0.67; *p* < 0.001), *LIAS* (HR = 0.61; 95%CI = 0.45–0.83; *p* = 0.002), *DLD* (HR = 0.53; 95%CI = 0.39–0.72; *p* < 0.001), *DLAT* (HR = 0.46; 95%CI = 0.33–0.63; *p* < 0.001), *PDHA1* (HR = 0.63; 95%CI = 0.46–0.85; *p* = 0.003), *PDHB* (HR = 0.57; 95%CI = 0.42–0.78; *p* < 0.001), *MTF1* (HR = 0.59; 95%CI = 0.43–0.80; *p* = 0.001), *GLS* (HR = 0.65; 95%CI = 0.48–0.88; *p* = 0.005), and *CDKN2A* (HR = 1.49; 95%CI = 1.10–2.02; *p* = 0.011) (Figure 1B).

**TABLE 1:** Characteristics of training, testing, and total cohort.

Clinical features	Type	Total (N = 518)	Testing cohort (N = 154)	Training cohort (N = 364)	*p*-value
Age	≤65	329 (63.51%)	99 (64.29%)	230 (63.19%)	0.8905
Age	>65	189 (36.49%)	55 (35.71%)	134 (36.81%)	
Stage	Stage I	261 (50.39%)	81 (52.6%)	180 (49.45%)	0.439
Stage	Stage II	58 (11.2%)	20 (12.99%)	38 (10.44%)	
Stage	Stage III	116 (22.39%)	34 (22.08%)	82 (22.53%)	
Stage	Stage IV	83 (16.02%)	19 (12.34%)	64 (17.58%)	
Gender	Female	175 (33.78%)	45 (29.22%)	130 (35.71%)	0.1846
Gender	Male	343 (66.22%)	109 (70.78%)	234 (64.29%)	
T	T1	267 (51.54%)	82 (53.25%)	185 (50.82%)	0.6336
T	T2	70 (13.51%)	24 (15.58%)	46 (12.64%)	
T	T3	170 (32.82%)	45 (29.22%)	125 (34.34%)	
T	T4	11 (2.12%)	3 (1.95%)	8 (2.2%)	
N	N0	230 (44.4%)	69 (44.81%)	161 (44.23%)	1
N	N1	15 (2.9%)	4 (2.6%)	11 (3.02%)	
N	Unknown	273 (52.7%)	81 (52.6%)	192 (52.75%)	
M	M0	414 (79.92%)	131 (85.06%)	283 (77.75%)	0.1085
M	M1	78 (15.06%)	17 (11.04%)	61 (16.76%)	
M	Unknown	26 (5.02%)	6 (3.9%)	20 (5.49%)	

### 3.3 Clinicopathological features

We compared the expression of cuproptosis-related genes at different clinical stages using the Wilcoxon rank-sum test. *FDX1*, *LIAS*, *DLD*, *DLAT*, *PDHA1*, *PDHB*, *MTF1*, and *GLS* were highly expressed in T1 compared with T3, and *CDKN2A* was expressed at lower levels in T1 than in T3 (Supplementary Figure S1). Compared to N1, *FDX1* and *LIAS* were highly expressed in N0 (Supplementary Figure S2). *FDX1, LIAS, DLD, DLAT, PDHA1, PDHB, MTF1*, and *GLS* were highly expressed in M0 compared with M1, and *CDKN2A* was expressed at lower levels in M0 than in M1 (Supplementary Figure S3). *FDX1, LIAS, DLD, DLAT, PDHA1, PDHB*, and *MTF1* were highly expressed in Stage 1 compared to Stage 3 and 4, and *CDKN2A* had lower expression in Stage 1 compared to Stage 3 and 4 (Supplementary Figure S4).

### 3.4 GO and KEGG

**FIGURE 2:**
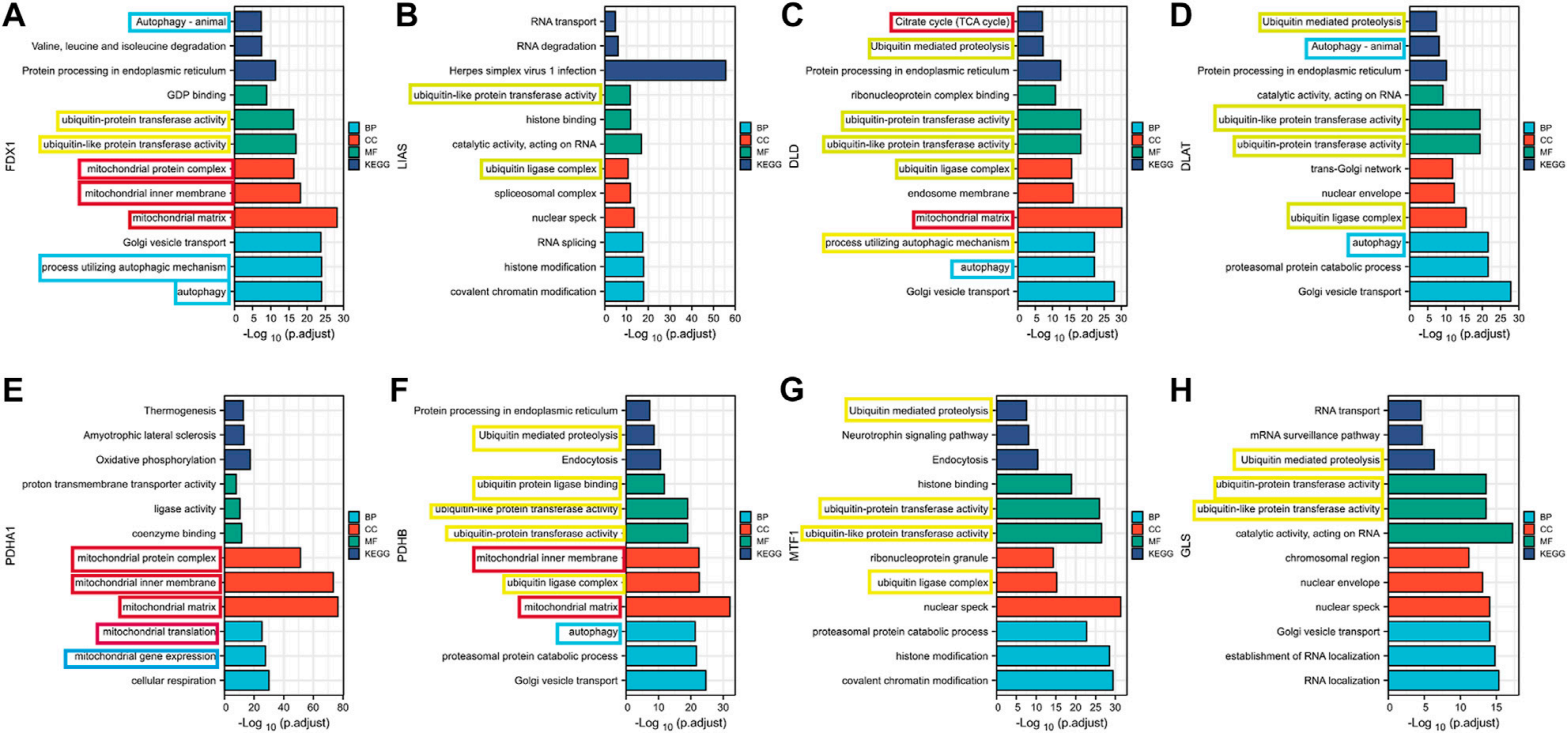
Results of GO and KEGG enrichment analysis of cuproptosis co-expression genes **(A)** FDX1; **(B)** LIAS **(C)** DLD; **(D)** DLAT; **(E)** PDHA1; **(F)** PDHB; **(G)**MTF1; **(H)** GLS.

For significant prognosis-related cuproptosis-related genes, we performed gene co-expression analysis in TCGA tumor patients and set the absolute value of the correlation coefficient to greater than 0.4 with a *p*-value less than 0.001 to obtain the co-expression genes. For co-expression genes, we performed GO and KEGG enrichment analyses and sorted them by *p* values (Figure 2). We found that co-expression genes were significantly enriched in the mitochondria during cell localization. The TCA cycle is thought to be associated with cancer progression, the site of biological processes in the mitochondria. This is consistent with Tsvetkov et al. (2022)’s view that Cu causes cell death by influencing the TCA cycle. The enrichment analysis results showed that co-expression of genes is correlated with autophagy and ubiquitin-mediated proteolysis, which provides a research direction for further exploration of the mechanism of cuproptosis.

### 3.5 Construction of the 4-CRGs risk signature

**FIGURE 3:**
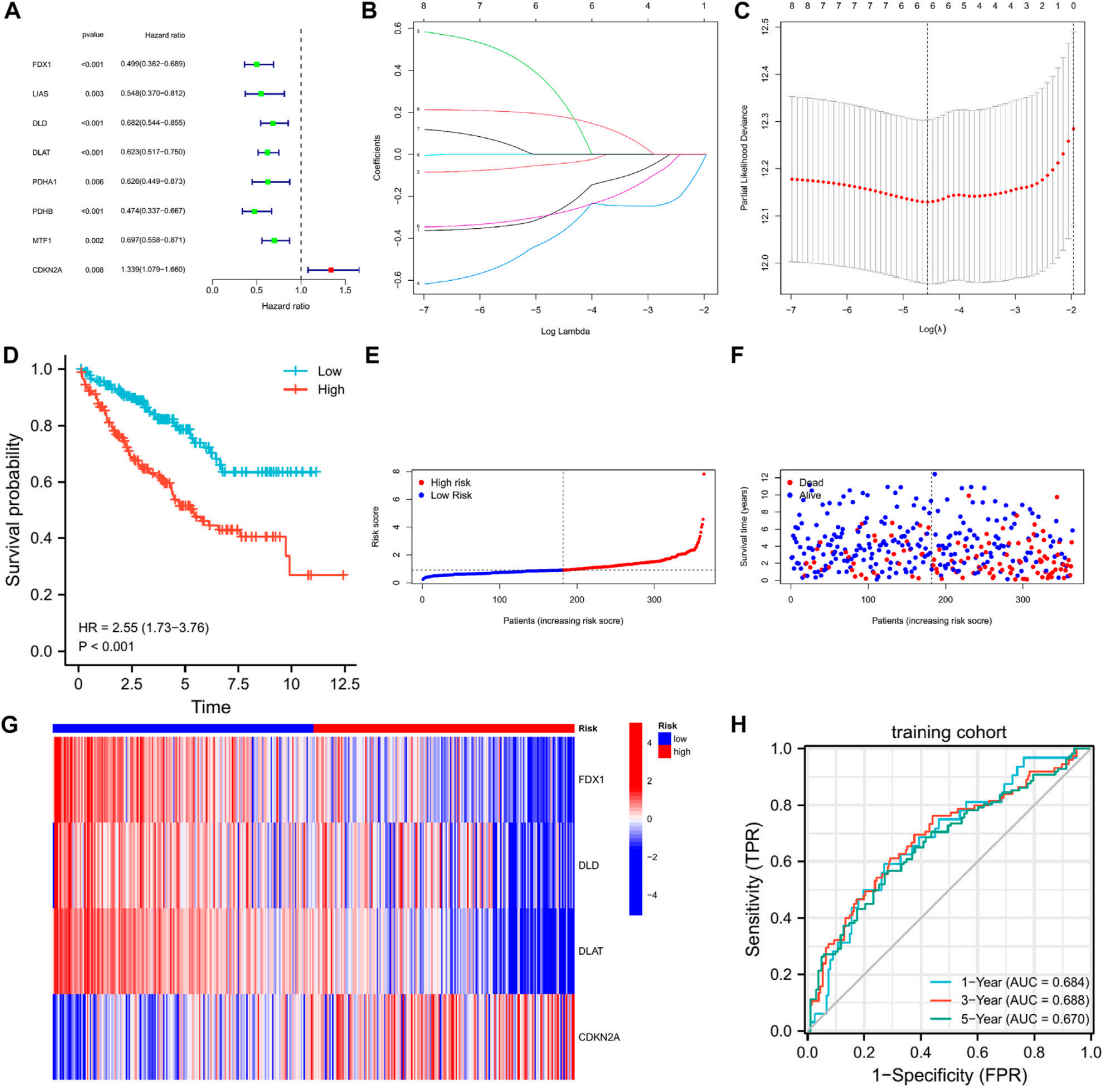
The 4-CRGs risk signature in the training cohort. **(A)** The CRGs with prognostic values were assessed by the univariate Cox proportional hazards regression model in the training cohort. **(B,C)** The selection of CRGs for risk signature by LASSO analysis in the training cohort. **(D)** K-M curves for OS in the training cohort. **(E–G)** The risk score, survival status, and heatmap of 4 CRGs in the training cohort. **(H)** Time-dependent ROC curves for OS in the training cohort.

We found no significant differences between the training and testing cohorts in terms of age, sex, or TNM staging (Table 1). In the training cohort, univariate Cox regression analysis yielded eight cuproptosis-related genes that were significantly associated with prognosis (Figure 3A). Using lasso regression method, six optimal variables were obtained from the above 8 cuproptosis-prognostic-related gene (Figures 3B,C). By Cox regression analysis, the signature was finally established: risk score = EXP FDX1 ∗ −0.499501220246694 + EXP DLD ∗ −0.59322127824406 + EXP DLAT ∗ −0.659153532219121 + EXP CDKN2A ∗ 0.199116740963518. The KM curve showed that the prognosis of the high-risk group was worse than that of the low-risk group (Figure 3D, log-rank *p* < 0.001; HR = 2.55, 95%CI = 1.73–3.76). ROC curves were used to assess the accuracy of the established models in predicting overall survival (OS) in patients with CCRCC. As shown in Figure 3H, the AUC values at 1, 3, and 5 years were 0.684, 0.688, and 0.670, respectively, indicating the robustness and accuracy of the model in predicting patient prognosis.

### 3.6 Validation of the 4-CRGs risk signature and validation of differential expression of *FDX1*, *DLD*, *DLAT*, and *CDKN2A* in CCRCC

**FIGURE 4:**
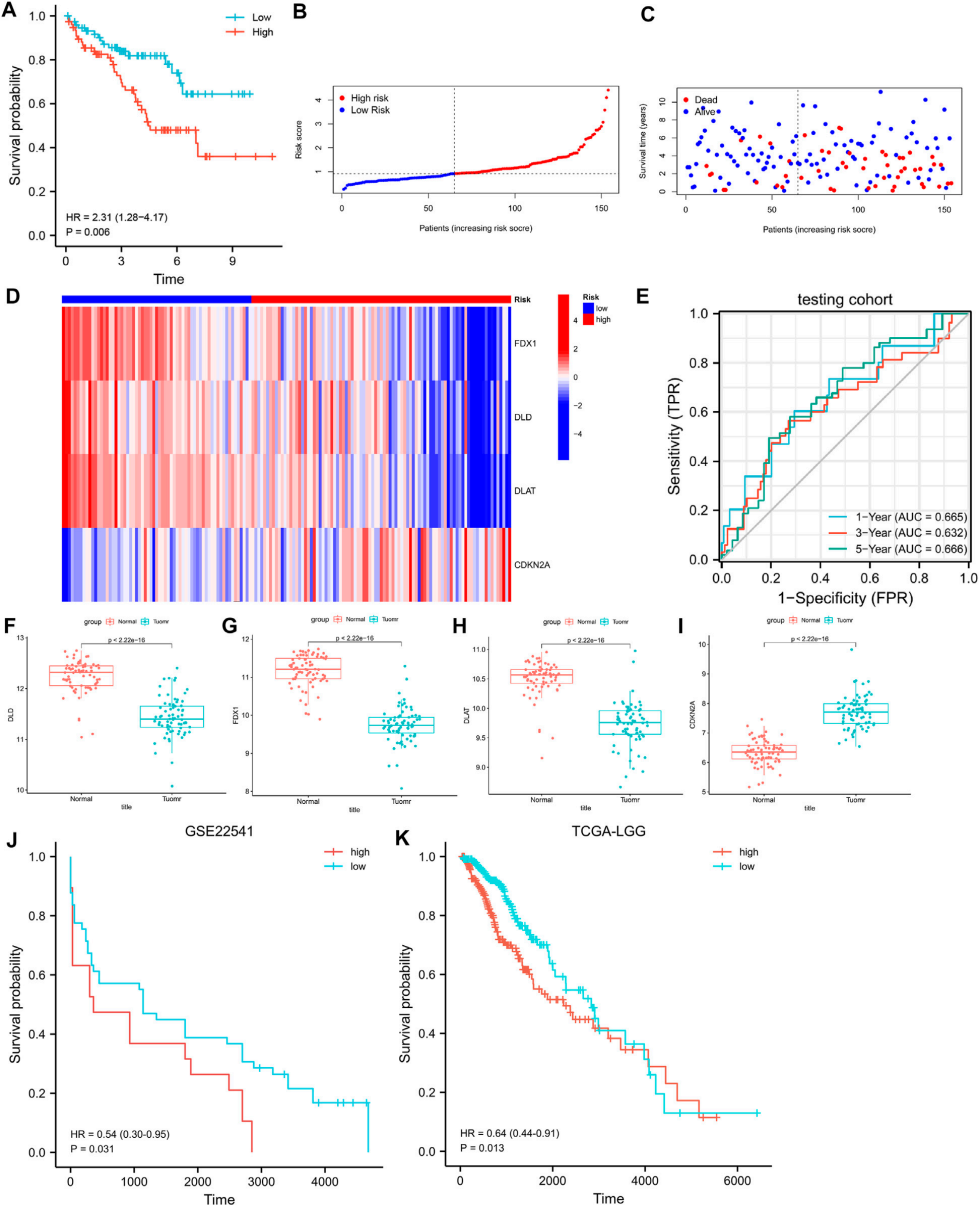
**(A)** K-M curves for OS in the testing cohort. **(B–D)** The risk score, survival status, and heatmap of 4 CRGs in the testing cohort. **(E)** Time-dependent ROC curves for OS in the testing cohort. **(F–I)** Differential expression of *FDX1*, *DLD*, *DLAT*, and *CDKN2A* in the GSE53757 dataset. **(J)** K-M curves for PFS in the GSE22541. **(K)** K-M curves for OS in the LGG.

In the testing cohort, the KM curve showed that the prognosis of the high-risk group was worse than that of the low-risk group (Figure 4A, log-rank *p* = 0.006; HR = 2.31, 95%CI = 1.28–4.17). ROC curves were used to assess the accuracy of the established models in predicting OS in patients with CCRCC. As shown in Figure 4E, the AUC values at 1, 3, and 5 years were 0.665, 0.632, and 0.666, respectively, indicating the robustness and accuracy of the model in predicting patient prognosis.

We validated the differences in the expression of *FDX1*, *DLD*, *DLAT*, and *CDKN2A* between CCRCC and normal samples using GSE53757. The results showed that *FDX1*, *DLD*, and *DLAT* exhibited low expression in CCRCC, whereas *CDKN2A* was highly expressed in CCRCC (Figures 4F–I). This is consistent with the results obtained from the TCGA dataset. We further verify the above prediction method in external data cohorts” GSE22541” and “TCGA-LGG”. In GSE22541 validation cohort, we divided the CCRCC patients into high-risk and low-risk groups based on the risk score. Survival comparison showed that low-risk group had significantly better prognosis outcome than high-risk group (Figure 4J). In addition, In TCGA-LGG validation cohort, we also found that based on the high and low-risk groups divided by risk score. Different groups have significantly different prognostic outcomes (Figure 4K). This demonstrates the generalization power of cuproptosis-related signature and has some value for the prediction of other cancers.

### 3.7 Nomogram and decision curve analysis

**FIGURE 5:**
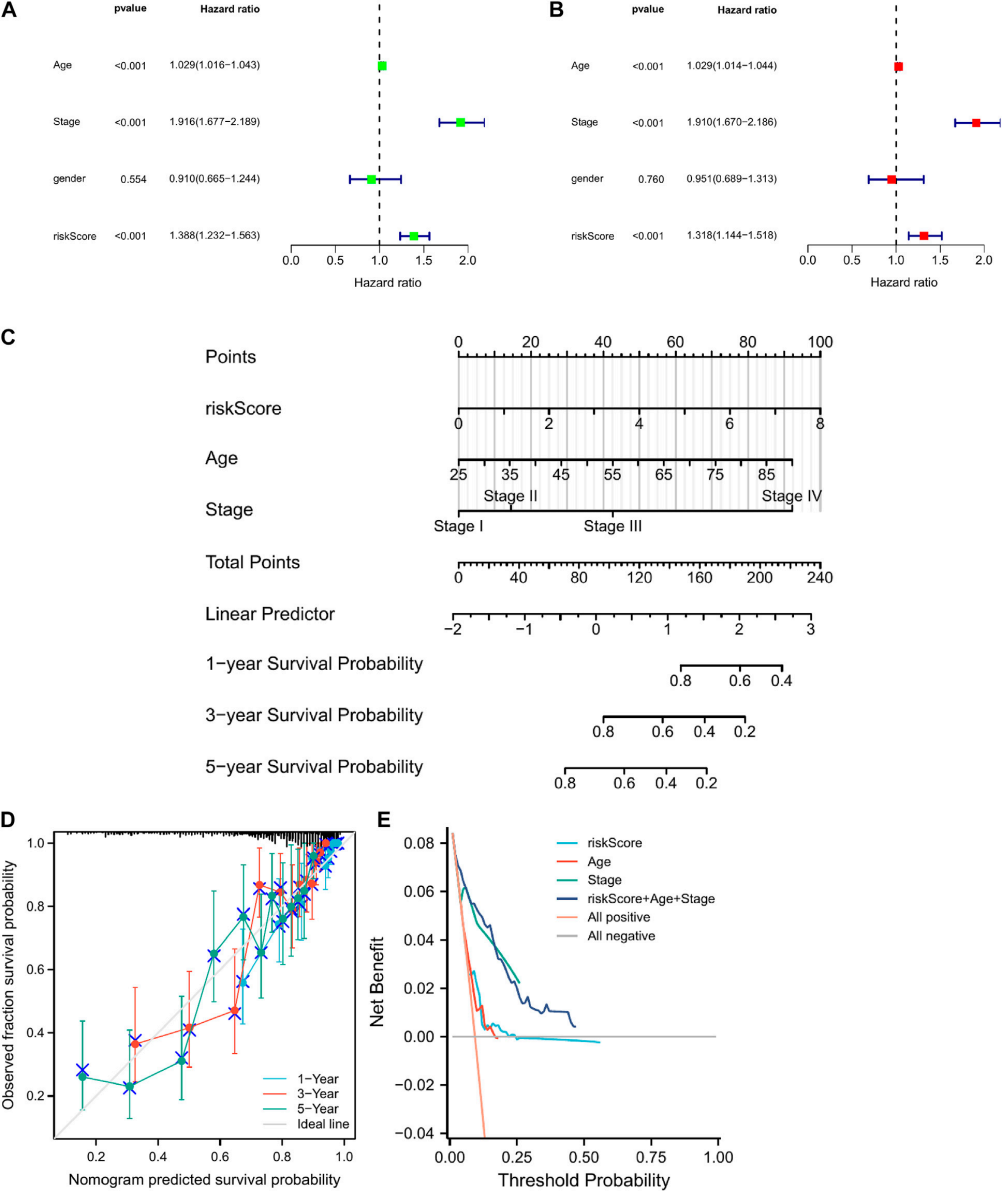
The construction of a nomogram for predicting survival. **(A,B)** Screening the independent predictors for OS in KIRC by univariate and multivariate Cox proportional hazards regression model. **(D)** A nomogram including risk score and clinicopathological features was constructed to predict 1/3/5-year OS. **(C)** The calibration plots for predicting 1/3/5-year OS are based on the CRGs nomogram. **(E)** Decision curve analysis (DCA) for the evaluation of the net benefits of CRGs and nomogram.

Univariate and multivariate Cox regression analyses showed that risk score, stage, and age were prognostic predictors of TGGA-KIRC (Figures 5A,B). Nomograms are widely used for the prognostic assessment of tumors. Various clinical features have prognostic value in clinical practice. Therefore, we established a nomogram containing multiple clinicopathological characteristics and risk scores. The scores for each variable were calculated and combined to predict the prognosis of patients with CCRCC (Figure 5C). DCA (Figure 5E) also proved that the nomogram combined with various clinical features had a better clinical application value.

### 3.8 Immune function and immune infiltrates

**FIGURE 6:**
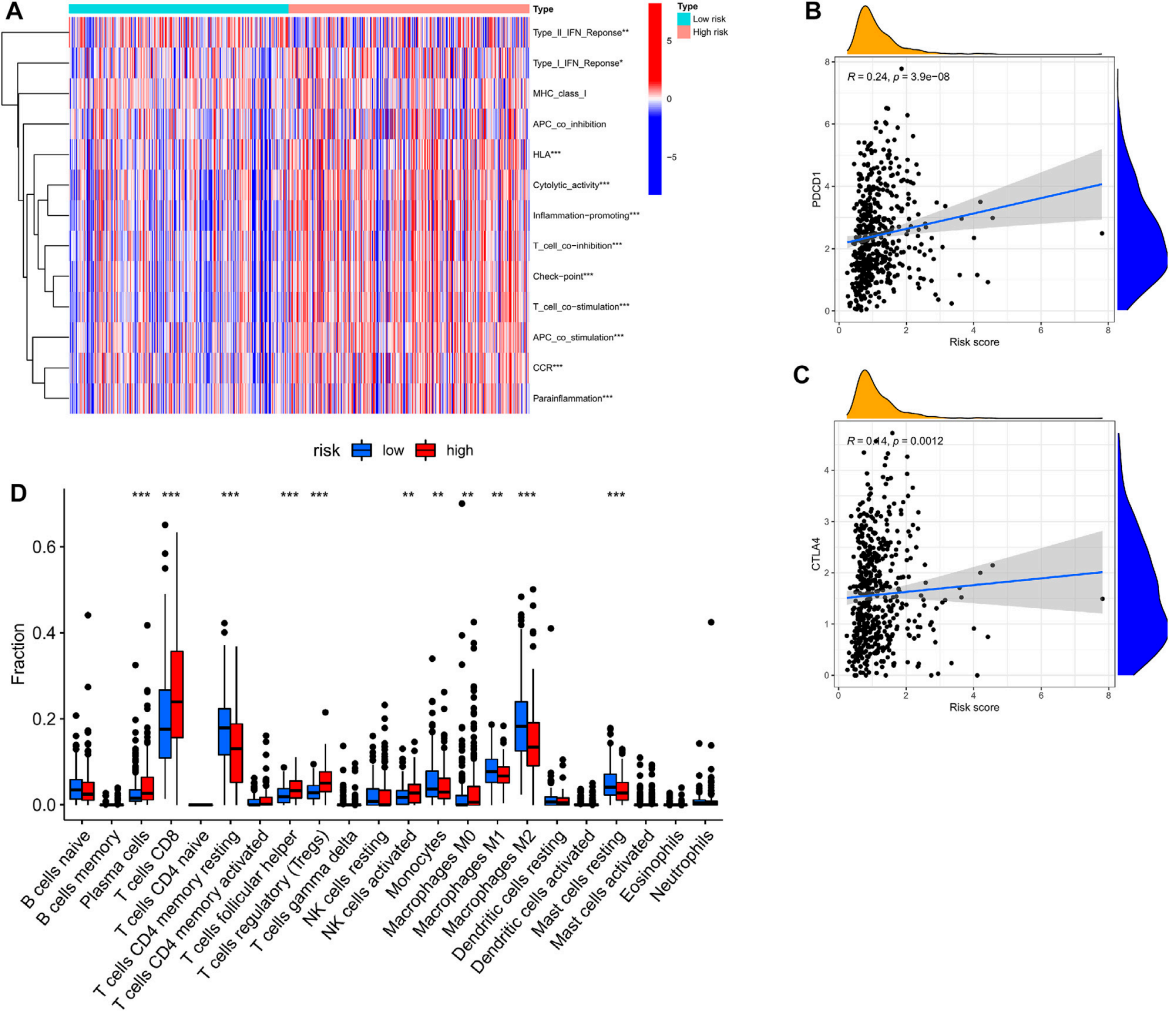
Correlation of CRGs signature with immunity. **(A)** Heatmap of the scores of immune-related functions between different risk groups. **(B,C)** The association between risk score and expression of PDCD1 or CTLA4 **(D)** Boxplots comparing the scores of immune cells between different risk groups. *p < 0.05; **p < 0.01, ***p < 0.001.

We used the CIBERSORT algorithm to estimate differences between 22 tumor-infiltrating immune cells between the low- and high-risk groups. Figure 6D shows that plasma cells, T cells CD8, T cells, regulatory T cells (Tregs), and activated NK cells were more enriched in high-risk groups, while naive B cells, T cells, CD4 memory monocytes, macrophages M0, macrophages M1, and macrophages M2 were more enriched in the low-risk group. This indicates that there are differences in immune cell infiltration in different risk groups, suggesting that cuproptosis -related genes are closely related to immune cell infiltration.

**FIGURE 7:**
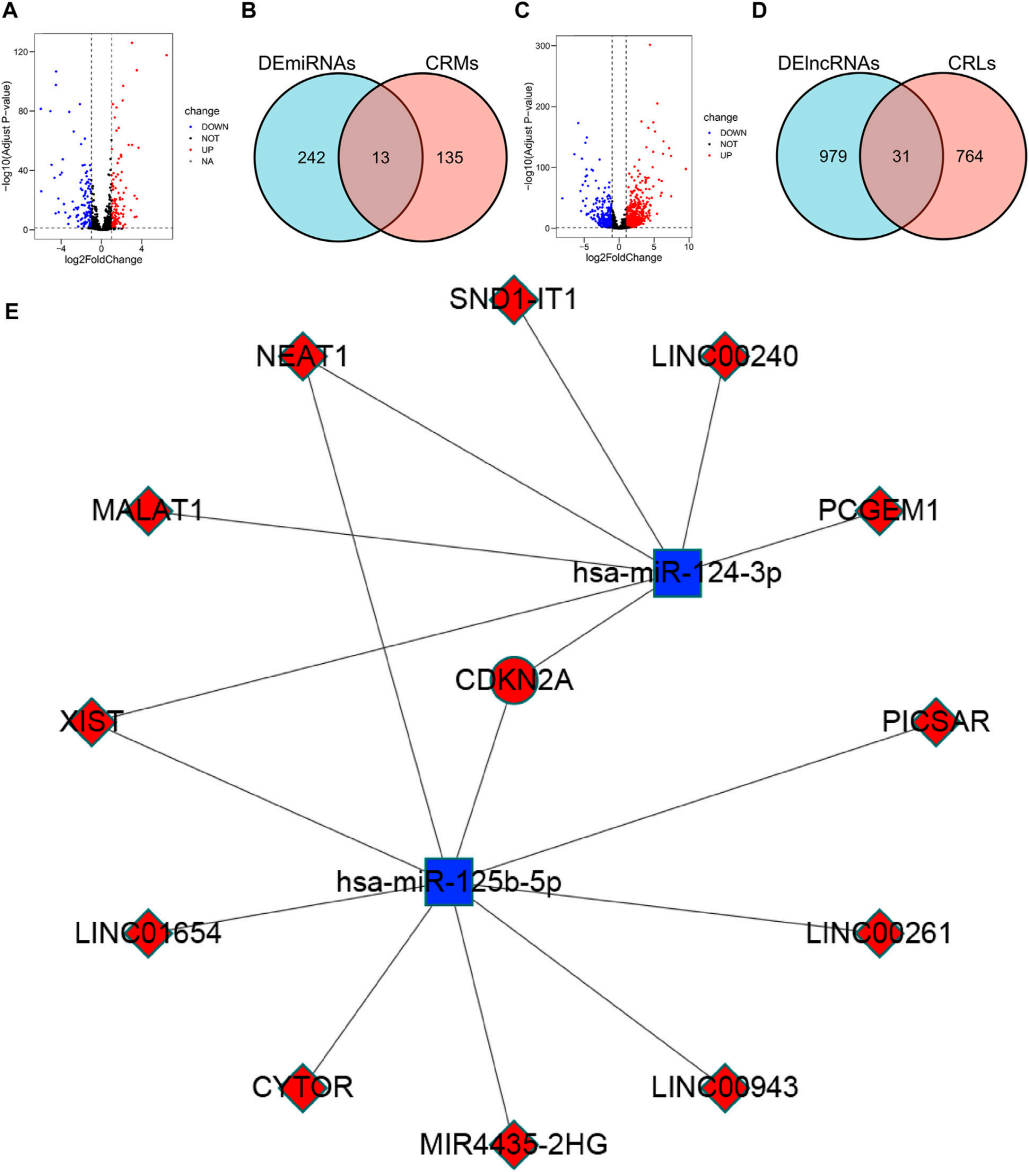
Construction of the ceRNA network. **(A)** Volcano plot of 255 differentially expressed miRNAs. **(B)** Venn diagram of the intersection of DEmiRNAs and CRMs. **(C)** Volcano plot of 1010 differentially expressed lncRNAs. **(D)** Venn diagram of the intersection of DElncRNAs and CRLs. **(E)** ceRNA network associated with cuproptosis: red indicates up-regulation in CCRCC, blue indicates down-regulation in CCRCC.

The ssGSEA method was applied to KIRC patients in the high- and low-risk groups to assess the differences in immune function between the high- and low-risk groups. Figure 7A shows that Type-I-IFN-Response, HLA, Cytolytic activity, Inflammation-promoting, T-cell-co-inhibition, Checkpoint, T-cell-co-stimulation, APC-co-stimulation, CCR, and parainflammation were upregulated in the high-risk group, suggesting that cuproptosis-related genes are involved in immune regulation.

We further investigated the links between *FDX1*, *DLD*, *DLAT*, and *CDKN2A* and the TIL gene markers in the TIMER database (Supplementary Material S1). *DLD* was strongly correlated with STAT3 (rho = 0.419), STAT5B (rho = 0.47), and CD4 (rho = −0.401) (Supplementary Figure S5). There was a strong correlation between *DLAT* and TGFBR2 (rho = 0.49), STAT3 (rho = 0.501), AHR (rho = 0.449), STAT5B (rho = 0.571), MRC1 (rho = 0.437), CD7 (rho = −0.417), and TGFBR2 (rho = 0.49) (Supplementary Figure S5). STAT3, STAT5B, and CD7 simultaneously show a strong correlation with *DLD* and *DLAT*, suggesting that STAT3, STAT5B, and CD7 may be associated with cuproptosis genes in an important link with levels of immune infiltration.

### 3.9 Immunomodulators and screening drugs

We explored the relationship between cuproptosis-related genes and immunomodulators associated with model building and found that *FDX1*, *DLD*, *DLAT*, *CDKN2A*, and immunostimulants were significantly associated. Specifically, high expression of *FDX1* was significantly associated with TNFRSF8 (Rho = −0.402), and high expression of *DLD* was significantly correlated with TNFRSF4 (Rho = −0.648), TNFRSF18 (Rho = −0.435), and TNFRSF25 (Rho = −0.6). *DLAT* was significantly correlated with TNFRSF4 (Rho = −0.522), TNFRSF8 (Rho = −0.436), TNFRSF18 (Rho = −0.557), TNFRSF25 (Rho = −0.642), CDKN2A, and TNFRSF18 (Rho = 0.398) (Supplementary Figure S6). Hence, *FDX1*, *DLD*, *DLAT*, and *CDKN2A* may play important roles in immune interactions and may be associated with tumor immune evasion.

We found that PDCD1 and CTLA4 (immune checkpoints) were elevated in patients with high-risk scores (Figures 6B,C). These discoveries suggested that patients of high-risk scores may be more sensitive to ICB therapy.

Finally, we predicted some drugs for tumor treatment using the pRRophetic R package and obtained some drugs that may show different sensitivities in patients in the high-risk and low-risk groups. Specifically, the low-risk group was more sensitive to AKT. inhibitor.VIII, AP.24534, AS601245, AUY922, axitinib, and AZ628, and the high-risk group were more sensitive to A.443654, ABT.888, AG.014699, AICAR, and AMG.706 (Supplementary Figure S7).

### 3.10 ceRNA

We analyzed DElncRNAs and DEmiRNAs between 541 KIRC samples and 72 normal samples and obtained a total of 255 DEmiRNAs, of which 129 were upregulated, 126 were downregulated; and 1010 DElncRNAs, of these, 777 were upregulated and 233 were downregulated. Using the miRTarBase database, 148 CRMs were identified. Thirteen CRDEMs were obtained by intersecting the CRMs with the DEmiRNAs. Subsequently, 795 CRLs were predicted using the starBase database, and 31 CRDELs were obtained by intersecting CRLs with DElncRNAs. Based on the existing theory that lncRNA inhibits the degradation of mRNA by miRNA through competitive binding of miRNA, we constructed a ceRNA network containing one mRNA, two miRNAs, and 12 lncRNAs (Figure 7E).

## 4 Discussion

We screened 10 copper ion carrier genes that are thought to be associated with cuproptosis. Nine out of 10 genes had differences in expression in CCRCC patients and non-tumor patients, and all 9 genes were valuable in assessing the prognosis of patients with CCRCC. We then looked for genes that were co-expressed with cuproptosis-related genes and found that cuproptosis-related genes may be associated with autophagy and ubiquitin-mediated proteolysis. Autophagy is associated with the survival of tumor cells but can either promote or inhibit apoptosis in different cellular contexts (Levy et al., 2017). Such context-dependent effects of autophagy are poorly understood; therefore, studying the relationship between apoptosis and autophagy may be a new research direction. Ubiquitin-mediated proteolysis is closely associated with cell proliferation. Studies have shown that the driving force of the cell cycle is the activation of cyclin-dependent kinases (CDKs), the activities of which are controlled by ubiquitin-mediated proteolysis of key regulators such as cyclins and CDK inhibitors (Nakayama and Nakayama, 2006). However, the link between cuproptosis and ubiquitin-mediated proteolysis needs to be experimentally confirmed. Using LASSO and multivariate Cox regression, we included four genes and constructed cuproptosis gene-related signatures containing *FDX1*, *DLD*, *DLAT*, and *CDKN2A*, of which *FDX1*, *DLAT*, and *CDKN2A* are correlated with CCRCC prognosis (Bian et al., 2022). Because *FDX1*, *DLD*, *DLAT*, and copper ion carriers are positively correlated, *CDKN2A* and copper ionophores are negatively correlated (Tsvetkov et al., 2022), which is consistent with the predictions. *FDX1* is a reductase that reduces cu^2+^ to cu^1+^ to promote cuproptosis (Tsvetkov et al., 2022). *FDX1* may modulate TP73 tumor suppressor through IRP2 to regulate tumor suppression (Zhang et al., 2020a), and *FDX1* may be a gene related to KIRC (Khouja et al., 2022). *DLD* is a homodimeric flavin-dependent enzyme that catalyzes NAD^+^-dependent oxidation of dihydrolipoamide and participates in the TCA cycle to convert pyruvate to acetyl-CoA (Fleminger and Dayan, 2021). *DLD* may destroy cancer cells by producing ROS and by chelation with DNA (Dayan et al., 2019). *DLAT* is the subunit E2 of the PDC complex in the TCA cycle (Goh et al., 2015). *DLAT* may promote apoptosis by influencing energy production. *CDKN2A* encodes the tumor suppressors p15 INK4b and p16 INK4a to inhibit CDK4 and CDK6, which prevent pRB phosphorylation and block cell cycle progression (Hannou et al., 2015). *CDKN2A* mutations may play a role in renal cancer metastasis by influencing the expression of p16/p14 (Sun et al., 2021). In summary, cuproptosis-related genes may play important roles in CCRCC.

CCRCC is an immunogenic tumor whose tumor immune microenvironment has many different immune cells infiltrates with various immunomodulatory molecules, which may have a significant impact on the prognosis of patients, as well as the effect of immunotherapy (Díaz-Montero et al., 2020). The cytolytic activity index (CYT) in CCRCC is the highest among 18 human cancers (Rooney et al., 2015), and spontaneous regression in 1% of patients are considered immune-mediated (Janiszewska et al., 2013). CD8^+^ T cells play an important role in tumor immunity, and their anti-tumor activity is the basis of ICI therapy (Şenbabaoğlu et al., 2016). Activated CD8^+^ T cells have a significant positive effect on the prognosis of some tumor patients, such as those with early colon cancer (Pagès et al., 2005; Galon et al., 2006). However, for CCRCC, infiltration of CD8^+^ T cells is associated with a high tumor grade and poor prognosis (Díaz-Montero et al., 2020). Our study showed that the high-risk group divided by the cuproptosis-related signature had a significant increase in the infiltration of CD8^+^ T cells compared with the low-risk group, which is consistent with most studies. TILs that do not mediate anti-tumor function may be associated with Tregs (Díaz-Montero et al., 2020; Hah and Koo, 2021). We found that the Treg infiltration levels were significantly higher in the high-risk group than in the low-risk group. STAT5B, a marker gene for Tregs, is highly correlated with the *DLD* and *DLAT* genes involved in signature construction and may be the key gene mediating elevated Treg infiltration levels in the high-risk group. In addition, immune checkpoint molecules are also important factors that block CD8^+^ T cells from exerting anti-tumor effects (Díaz-Montero et al., 2020). We found that both the most important immune checkpoint molecules, PDCD1 and CTLA-4, were positively correlated with the risk scores. In addition, we also found that the high-risk group had a higher TIDE value, which also suggested that the high-risk group was more likely to develop immune escape.

To make the cuproptosis-related signature more clinically relevant, we screened some of the drugs with different sensitivities in the high-risk and low-risk groups. Patients in the low-risk group had greater sensitivity to axitinib, an anti-VEGF-targeted drug used for the treatment of metastatic RCC (Hsieh et al., 2017).

The ceRNA hypothesis proposes that lncRNAs, as competing endogenous RNAs, regulate mRNA expression by competing for shared miRNAs (Karreth and Pandolfi, 2013). Specifically, upregulated lncRNA can competitively bind to miRNA, causing miRNA expression to be downregulated to inhibit the degradation of mRNA by miRNA and promote mRNA expression. We constructed a ceRNA network containing one upregulated mRNA, two down-regulated miRNAs, and 12 up-regulated lncRNAs. In the ceRNA network, we constructed CDKN2A, an upregulated mRNA whose high expression is thought to be associated with poor prognosis in CCRCC. Hsa-mir-124-3p, a downregulated miRNA predicted to bind to CDKN2A, is considered a key miRNA in CCRCC, inhibiting tumor migration, invasion, and proliferation (Butz et al., 2015). XIST, MALAT1, NEAT1, and LINC00240 up-regulated lncRNA were predicted to bind to hsa-mir-124-3p and promoted the proliferation and metastasis of other cancers by modulating hsa-mir-124-3p, but there are no related studies in CCRCC (Feng et al., 2016; Liu et al., 2018a; Xiao et al., 2019; Zhang et al., 2020b). In cervical cancer, Hsa-mir-125b-5p expression was downregulated, and CDKN2A expression was upregulated, suggesting that hsa-miR-125a-5p-CDKN2A is a possible ceRNA network (Wang et al., 2021). Hsa-mir-125b-5p was also found to be downregulated in bladder (Canturk et al., 2014) and prostate cancer (Lin et al., 2020). XIST, a lncRNA, has been shown to promote the progression of various cancers through its high expression (Liu et al., 2018b; Liu et al., 2019; Ning et al., 2021; Zheng et al., 2021). We found that the upregulated lncRNA XIST targets both hsa-mir-124-3p and hsa-miR-125b-5p and is positively correlated with CDKN2A. A XIST-hsa-mir-124-3p/hsa-miR-125b-5p-CDKN2A ceRNA network may exist in CCRCC and play an important role in its prognosis and development.

However, this study has some limitations and deficiencies. First, our study was retrospective, and prospective studies are needed to confirm these findings. Second, our conclusions were all obtained by data analysis and need to be further confirmed by experiments.

## Data Availability

The datasets presented in this study can be found in online repositories. The names of the repository/repositories and accession number(s) can be found below: https://www.ncbi.nlm.nih.gov/geo/, GSE53757.

## References

[B1] Allensworth J. L. Evans M. K. Bertucci F. Aldrich A. J. Festa R. A. Finetti P. (2015). Disulfiram (DSF) acts as a copper ionophore to induce copper-dependent oxidative stress and mediate anti-tumor efficacy in inflammatory breast cancer. Mol. Oncol. 9 (6), 1155–1168. 10.1016/j.molonc.2015.02.007 25769405 PMC4493866

[B2] Bian Z. Fan R. Xie L. (2022). A novel cuproptosis-related prognostic gene signature and validation of differential expression in clear cell renal cell carcinoma. Genes. (Basel) 13 (5), 851. 10.3390/genes13050851 35627236 PMC9141858

[B3] Butz H. Szabo P. M. Khella H. W. Z. Nofech-Mozes R. Patocs A. Yousef G. M. (2015). miRNA-target network reveals miR-124as a key miRNA contributing to clear cell renal cell carcinoma aggressive behaviour by targeting CAV1 and FLOT1. Oncotarget 6 (14), 12543–12557. 10.18632/oncotarget.3815 26002553 PMC4494957

[B4] Canturk K. M. Ozdemir M. Can C. Öner S. Emre R. Aslan H. (2014). Investigation of key miRNAs and target genes in bladder cancer using miRNA profiling and bioinformatic tools. Mol. Biol. Rep. 41 (12), 8127–8135. 10.1007/s11033-014-3713-5 25189652

[B5] da Silva D. A. De Luca A. Squitti R. Rongioletti M. Rossi L. Machado C. M. L. (2022). Copper in tumors and the use of copper-based compounds in cancer treatment. J. Inorg. Biochem. 226, 111634. 10.1016/j.jinorgbio.2021.111634 34740035

[B6] Dayan A. Fleminger G. Ashur-Fabian O. (2019). Targeting the Achilles' heel of cancer cells via integrin-mediated delivery of ROS-generating dihydrolipoamide dehydrogenase. Oncogene 38 (25), 5050–5061. 10.1038/s41388-019-0775-9 30872792

[B7] De Luca A. Barile A. Arciello M. Rossi L. (2019). Copper homeostasis as target of both consolidated and innovative strategies of anti-tumor therapy. J. Trace Elem. Med. Biol. 55, 204–213. 10.1016/j.jtemb.2019.06.008 31345360

[B8] Denoyer D. Masaldan S. La Fontaine S. Cater M. A. (2015). Targeting copper in cancer therapy: 'Copper that cancer. Metallomics 7 (11), 1459–1476. 10.1039/c5mt00149h 26313539

[B9] Díaz-Montero C. M. Rini B. I. Finke J. H. (2020). The immunology of renal cell carcinoma. Nat. Rev. Nephrol. 16 (12), 721–735. 10.1038/s41581-020-0316-3 32733094

[B10] Feng T. Shao F. Wu Q. Zhang X. Xu D. Qian K. (2016). miR-124 downregulation leads to breast cancer progression via LncRNA-MALAT1 regulation and CDK4/E2F1 signal activation. Oncotarget 7 (13), 16205–16216. 10.18632/oncotarget.7578 26918449 PMC4941308

[B11] Fleminger G. Dayan A. (2021). The moonlighting activities of dihydrolipoamide dehydrogenase: Biotechnological and biomedical applications. J. Mol. Recognit. 34 (11), e2924. 10.1002/jmr.2924 34164859

[B12] Galon J. Costes A. Sanchez-Cabo F. Kirilovsky A. Mlecnik B. Lagorce-Pages C. (2006). Type, density, and location of immune cells within human colorectal tumors predict clinical outcome. Science 313 (5795), 1960–1964. 10.1126/science.1129139 17008531

[B13] Goh W. Q. Ow G. S. Kuznetsov V. A. Chong S. Lim Y. P. (2015). DLAT subunit of the pyruvate dehydrogenase complex is upregulated in gastric cancer-implications in cancer therapy. Am. J. Transl. Res. 7 (6), 1140–1151. 26279757 PMC4532746

[B14] Hah Y. S. Koo K. C. (2021). Immunology and immunotherapeutic approaches for advanced renal cell carcinoma: A comprehensive review. Int. J. Mol. Sci. 22 (9), 4452. 10.3390/ijms22094452 33923219 PMC8123195

[B15] Hannou S. A. Wouters K. Paumelle R. Staels B. (2015). Functional genomics of the CDKN2A/B locus in cardiovascular and metabolic disease: What have we learned from GWASs? Trends Endocrinol. Metab. 26 (4), 176–184. 10.1016/j.tem.2015.01.008 25744911

[B16] Hsieh J. J. Purdue M. P. Signoretti S. Swanton C. Albiges L. Schmidinger M. (2017). Renal cell carcinoma. Nat. Rev. Dis. Prim. 3, 17009. 10.1038/nrdp.2017.9 28276433 PMC5936048

[B17] Huang H. Y. miRTarBase Li J. Huang K. Y. Shrestha S. Hong H. C. (2020). miRTarBase 2020: updates to the experimentally validated microRNA-target interaction database. Nucleic Acids Res. 48 (1), D148–D154. 10.1093/nar/gkz896 31647101 PMC7145596

[B18] Janiszewska A. D. Poletajew S. Wasiutyński A. (2013). Spontaneous regression of renal cell carcinoma. Contemp. Oncol. 17 (2), 123–127. 10.5114/wo.2013.34613 PMC3685371 23788977

[B19] Karreth F. A. Pandolfi P. P. (2013). ceRNA cross-talk in cancer: when ce-bling rivalries go awry. Cancer Discov. 3 (10), 1113–1121. 10.1158/2159-8290.CD-13-0202 24072616 PMC3801300

[B20] Khouja H. I. Ashankyty I. M. Bajrai L. H. Kumar P. K. P. Kamal M. A. Firoz A. (2022). Multi-staged gene expression profiling reveals potential genes and the critical pathways in kidney cancer. Sci. Rep. 12 (1), 7240. 10.1038/s41598-022-11143-6 35508649 PMC9065671

[B21] Levy J. M. M. Towers C. G. Thorburn A. (2017). Targeting autophagy in cancer. Nat. Rev. Cancer 17 (9), 528–542. 10.1038/nrc.2017.53 28751651 PMC5975367

[B22] Li J. H. Liu S. Zhou H. Qu L. H. Yang J. H. (2014). starBase v2.0: decoding miRNA-ceRNA, miRNA-ncRNA and protein-RNA interaction networks from large-scale CLIP-Seq data. Nucleic Acids Res. 42, D92–D97. 10.1093/nar/gkt1248 24297251 PMC3964941

[B23] Li T. Fu J. Zeng Z. Cohen D. Li J. Chen Q. (2020). TIMER2.0 for analysis of tumor-infiltrating immune cells. Nucleic Acids Res. 48 (1), W509–W514. 10.1093/nar/gkaa407 32442275 PMC7319575

[B24] Lin Y. Miao Z. Zhang X. Wei X. Hou J. Huang Y. (2020). Identification of key MicroRNAs and mechanisms in prostate cancer evolution based on biomarker prioritization model and carcinogenic survey. Front. Genet. 11, 596826. 10.3389/fgene.2020.596826 33519899 PMC7844321

[B25] Liu H. Deng H. Zhao Y. Liang Y. (2018). LncRNA XIST/miR-34a axis modulates the cell proliferation and tumor growth of thyroid cancer through MET-PI3K-AKT signaling. J. Exp. Clin. Cancer Res. 37 (1), 279. 10.1186/s13046-018-0950-9 30463570 PMC6249781

[B26] Liu J. Yao L. Zhang M. Jiang J. Yang M. Wang Y. (2019). Downregulation of LncRNA-XIST inhibited development of non-small cell lung cancer by activating miR-335/SOD2/ROS signal pathway mediated pyroptotic cell death. Aging (Albany NY) 11 (18), 7830–7846. 10.18632/aging.102291 31553952 PMC6781979

[B27] Liu X. Liang Y. Song R. Yang G. Han J. Lan Y. (2018). Long non-coding RNA NEAT1-modulated abnormal lipolysis via ATGL drives hepatocellular carcinoma proliferation. Mol. Cancer 17 (1), 90. 10.1186/s12943-018-0838-5 29764424 PMC5953401

[B28] Motzer R. J. Escudier B. McDermott D. F. George S. Hammers H. J. Srinivas S. (2015). Nivolumab versus everolimus in advanced renal-cell carcinoma. N. Engl. J. Med. 373 (19), 1803–1813. 10.1056/NEJMoa1510665 26406148 PMC5719487

[B29] Motzer R. J. Penkov K. Haanen J. Rini B. Albiges L. Campbell M. T. (2019). Avelumab plus axitinib versus sunitinib for advanced renal-cell carcinoma. N. Engl. J. Med. 380 (12), 1103–1115. 10.1056/NEJMoa1816047 30779531 PMC6716603

[B30] Nabi S. Kessler E. R. Bernard B. Flaig T. W. Lam E. T. (2018). Renal cell carcinoma: A review of biology and pathophysiology. F1000Res. 7, 307. 10.12688/f1000research.13179.1 29568504 PMC5850086

[B31] Nakayama K. I. Nakayama K. (2006). Ubiquitin ligases: Cell-cycle control and cancer. Nat. Rev. Cancer 6 (5), 369–381. 10.1038/nrc1881 16633365

[B32] Nechushtan H. Hamamreh Y. Nidal S. Gotfried M. Baron A. Shalev Y. I. (2015). A phase IIb trial assessing the addition of disulfiram to chemotherapy for the treatment of metastatic non-small cell lung cancer. Oncologist 20 (4), 366–367. 10.1634/theoncologist.2014-0424 25777347 PMC4391770

[B33] Newman A. M. Liu C. L. Green M. R. Gentles A. J. Feng W. Xu Y. (2015). Robust enumeration of cell subsets from tissue expression profiles. Nat. Methods 12 (5), 453–457. 10.1038/nmeth.3337 25822800 PMC4739640

[B34] Ning D. Chen J. Du P. Liu Q. Cheng Q. Li X. (2021). The crosstalk network of XIST/miR-424-5p/OGT mediates RAF1 glycosylation and participates in the progression of liver cancer. Liver Int. 41 (8), 1933–1944. 10.1111/liv.14904 33909326

[B35] Pagès F. Berger A. Camus M. Sanchez-Cabo F. Costes A. Molidor R. (2005). Effector memory T cells, early metastasis, and survival in colorectal cancer. N. Engl. J. Med. 353 (25), 2654–2666. 10.1056/NEJMoa051424 16371631

[B36] Patard J.-J. Leray E. Rodriguez A. Rioux-Leclercq N. Guille F. Lobel B. (2003). Correlation between symptom graduation, tumor characteristics and survival in renal cell carcinoma. Eur. Urol. 44 (2), 226–232. 10.1016/s0302-2838(03)00216-1 12875943

[B37] Rini B. I. Plimack E. R. Stus V. Gafanov R. Hawkins R. Nosov D. (2019). Pembrolizumab plus axitinib versus sunitinib for advanced renal-cell carcinoma. N. Engl. J. Med. 380 (12), 1116–1127. 10.1056/NEJMoa1816714 30779529

[B38] Rooney M. S. Shukla S. A. Wu C. J. Getz G. Hacohen N. (2015). Molecular and genetic properties of tumors associated with local immune cytolytic activity. Cell. 160 (1-2), 48–61. 10.1016/j.cell.2014.12.033 25594174 PMC4856474

[B39] Safi R. Nelson E. R. Chitneni S. K. Franz K. J. George D. J. Zalutsky M. R. (2014). Copper signaling axis as a target for prostate cancer therapeutics. Cancer Res. 74 (20), 5819–5831. 10.1158/0008-5472.CAN-13-3527 25320179 PMC4203427

[B40] Şenbabaoğlu Y. Gejman R. S. Winer A. G. Liu M. Van Allen E. M. de Velasco G. (2016). Erratum to: Tumor immune microenvironment characterization in clear cell renal cell carcinoma identifies prognostic and immunotherapeutically relevant messenger RNA signatures. Genome Biol. 17 (1), 46. 10.1186/s13059-017-1180-8 27855702 PMC5114739

[B41] Shanbhag V. C. Gudekar N. Jasmer K. Papageorgiou C. Singh K. Petris M. J. (2021). Copper metabolism as a unique vulnerability in cancer. Biochim. Biophys. Acta. Mol. Cell. Res. 1868 (2), 118893. 10.1016/j.bbamcr.2020.118893 33091507 PMC7779655

[B42] Siegel R. L. Miller K. D. Jemal A. (2020). Cancer statistics. Ca. Cancer J. Clin. 70 (1), 7–30. 10.3322/caac.21590 31912902

[B43] Sonpavde G. Choueiri T. K. Escudier B. Ficarra V. Hutson T. E. Mulders P. F. (2012). Sequencing of agents for metastatic renal cell carcinoma: Can we customize therapy? Eur. Urol. 61 (2), 307–316. 10.1016/j.eururo.2011.10.032 22055147

[B44] Sun Q. Chen S. Hou Y. Wen X. Teng X. Zhang H. (2021). Mutant CDKN2A regulates P16/p14 expression by alternative splicing in renal cell carcinoma metastasis. Pathol. Res. Pract. 223, 153453. 10.1016/j.prp.2021.153453 34022680

[B45] Tibshirani R. (1997). The lasso method for variable selection in the Cox model. Stat. Med. 16 (4), 385–395. 10.1002/(sici)1097-0258(19970228)16:4<385::aid-sim380>3.0.co;2-3 9044528

[B46] Tsvetkov P. Coy S. Petrova B. Dreishpoon M. Verma A. Abdusamad M. (2022). Copper induces cell death by targeting lipoylated TCA cycle proteins. Science 375 (6586), 1254–1261. 10.1126/science.abf0529 35298263 PMC9273333

[B47] von Roemeling C. A. Radisky D. C. Marlow L. A. Cooper S. J. Grebe S. K. Anastasiadis P. Z. (2014). Neuronal pentraxin 2 supports clear cell renal cell carcinoma by activating the AMPA-selective glutamate receptor-4. Cancer Res. 74 (17), 4796–4810. 10.1158/0008-5472.CAN-14-0210 24962026 PMC4154999

[B48] Wang T. Zhang X. D. Hua K. Q. (2021). A ceRNA network of BBOX1-AS1-hsa-miR-125b-5p/hsa-miR-125a-5p-CDKN2A shows prognostic value in cervical cancer. Taiwan. J. Obstet. Gynecol. 60 (2), 253–261. 10.1016/j.tjog.2020.12.006 33678324

[B49] Xiao D. Cui X. Wang X. (2019). Long noncoding RNA XIST increases the aggressiveness of laryngeal squamous cell carcinoma by regulating miR-124-3p/EZH2. Exp. Cell. Res. 381 (2), 172–178. 10.1016/j.yexcr.2019.04.034 31071316

[B50] Zhang H. Chen D. Ringler J. Chen W. Cui Q. C. Ethier S. P. (2010). Disulfiram treatment facilitates phosphoinositide 3-kinase inhibition in human breast cancer cells *in vitro* and *in vivo* . Cancer Res. 70 (10), 3996–4004. 10.1158/0008-5472.CAN-09-3752 20424113 PMC3827685

[B51] Zhang J. Kong X. Zhang Y. Sun W. Wang J. Chen M. (2020). FDXR regulates TP73 tumor suppressor via IRP2 to modulate aging and tumor suppression. J. Pathol. 251 (3), 284–296. 10.1002/path.5451 32304229 PMC7748393

[B52] Zhang Y. Li X. Zhang J. Liang H. (2020). Natural killer T cell cytotoxic activity in cervical cancer is facilitated by the LINC00240/microRNA-124-3p/STAT3/MICA axis. Cancer Lett. 474, 63–73. 10.1016/j.canlet.2019.12.038 31904481

[B53] Zheng H. Zhang M. Ke X. Deng X. Li D. Wang Q. (2021). LncRNA XIST/miR-137 axis strengthens chemo-resistance and glycolysis of colorectal cancer cells by hindering transformation from PKM2 to PKM1. Cancer Biomark. 30 (4), 395–406. 10.3233/CBM-201740 33386794 PMC12499988

