# Supplementary figures and images for: The cuproptosis related genes signature predicts the prognosis and correlates with the immune status of clear cell renal cell carcinoma
Source: Front Genet. 2022 Dec 1;13:1061382. doi: 10.3389/fgene.2022.1061382 (PMC9751702; doi:10.3389/fgene.2022.1061382)

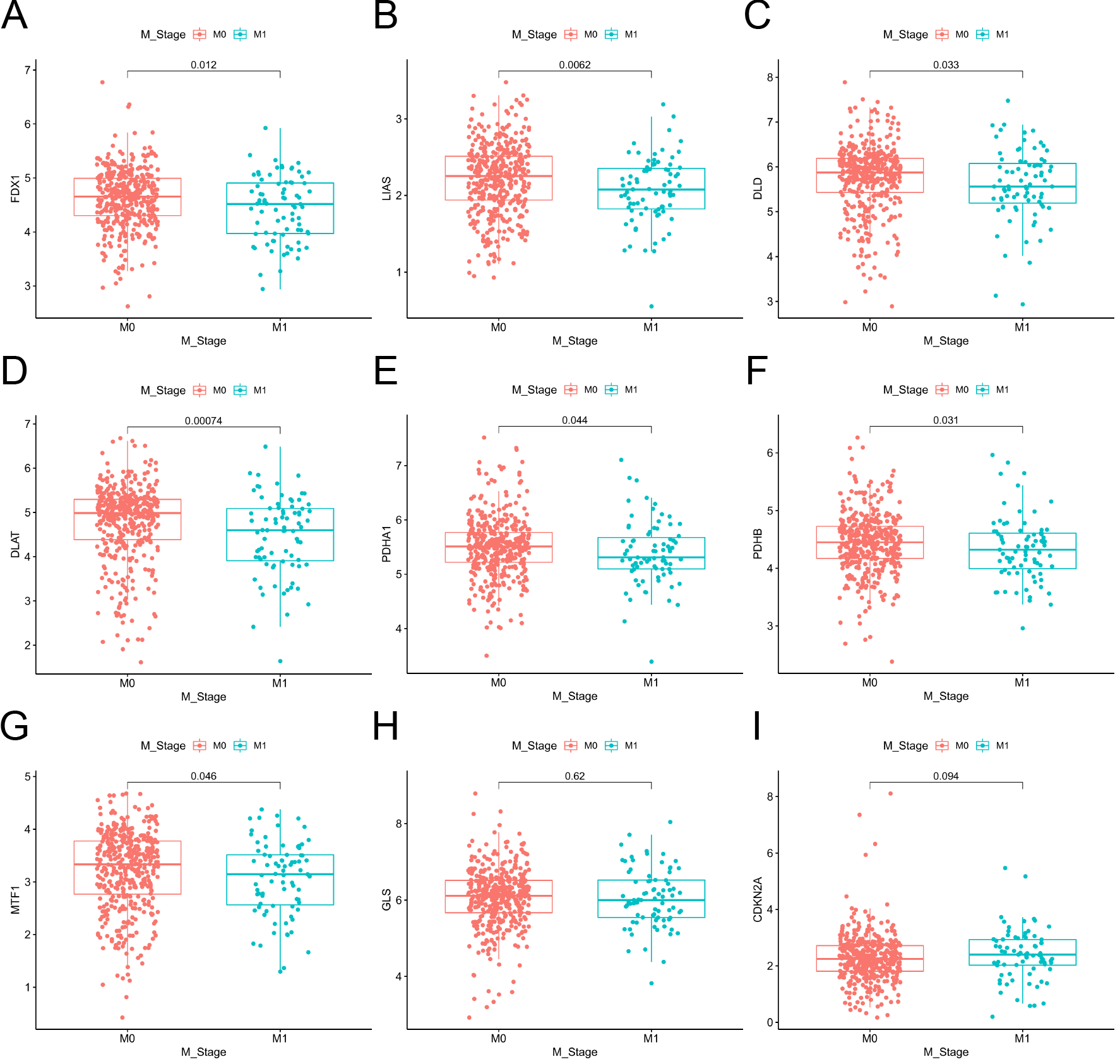

Supplement: Supplementary file 1 [file Image3.TIFF]

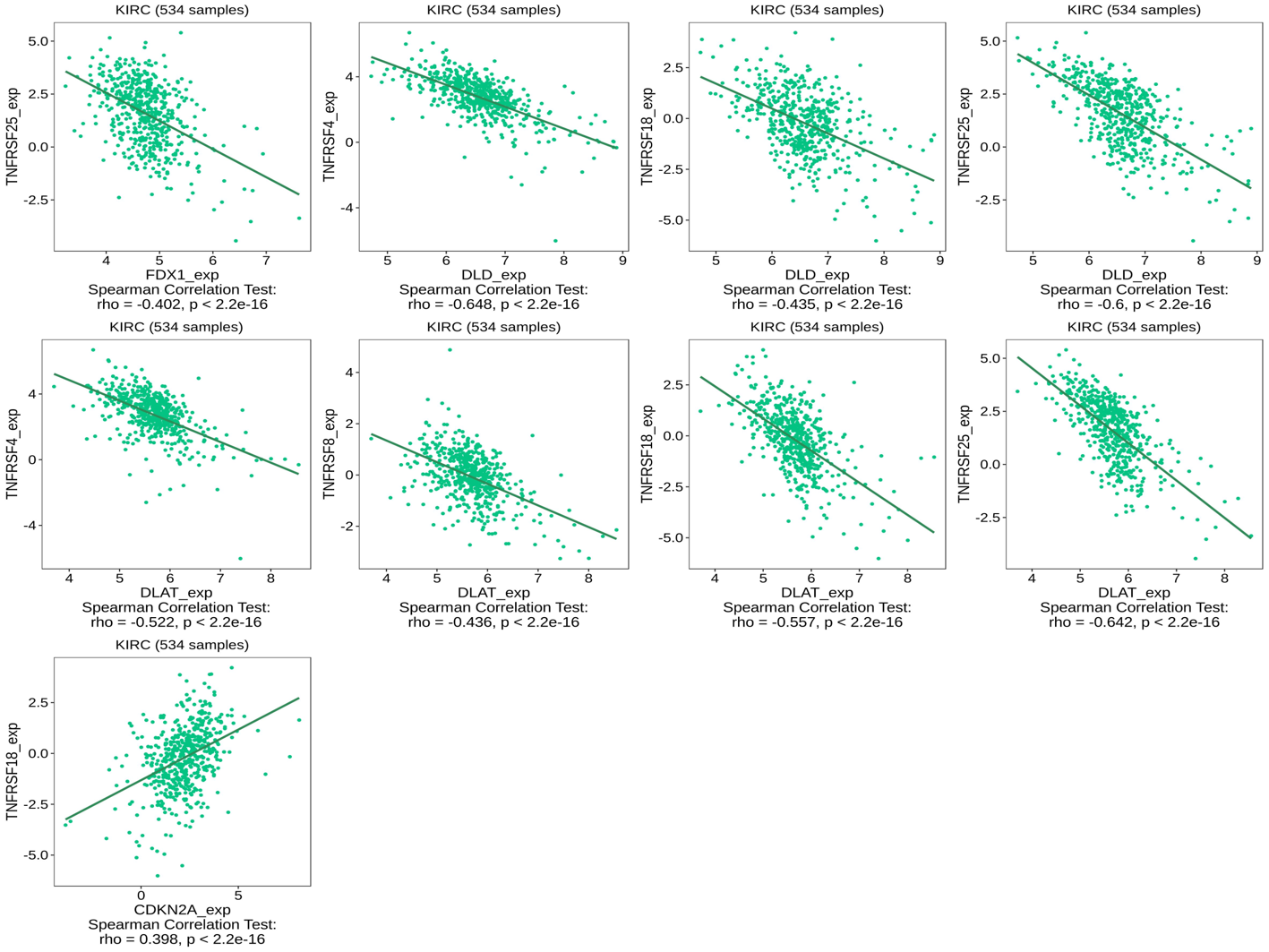

Supplement: Supplementary file 2 [file Image6.TIF]

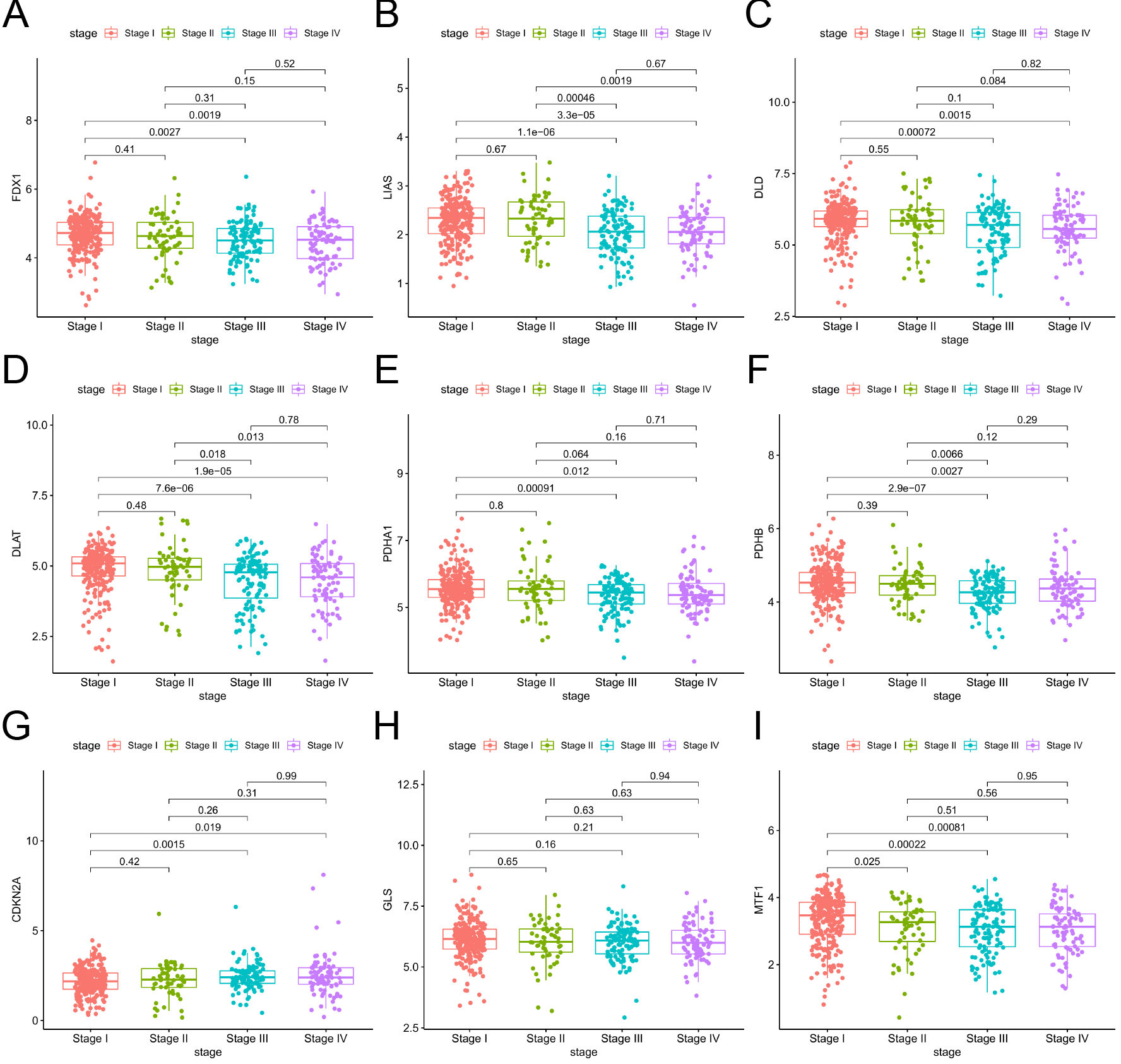

Supplement: Supplementary file 3 [file Image4.TIF]

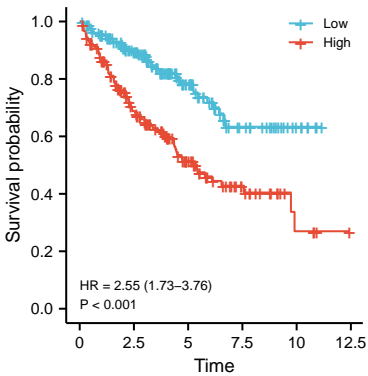

Supplement: Supplementary file 4 [file DataSheet1.ZIP › RAW.data/17.KM/tarinKM.pdf]

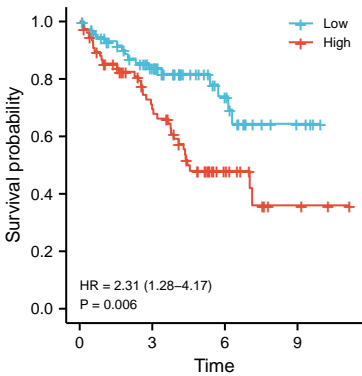

Supplement: Supplementary file 4 [file DataSheet1.ZIP › RAW.data/17.KM/testKM.pdf]

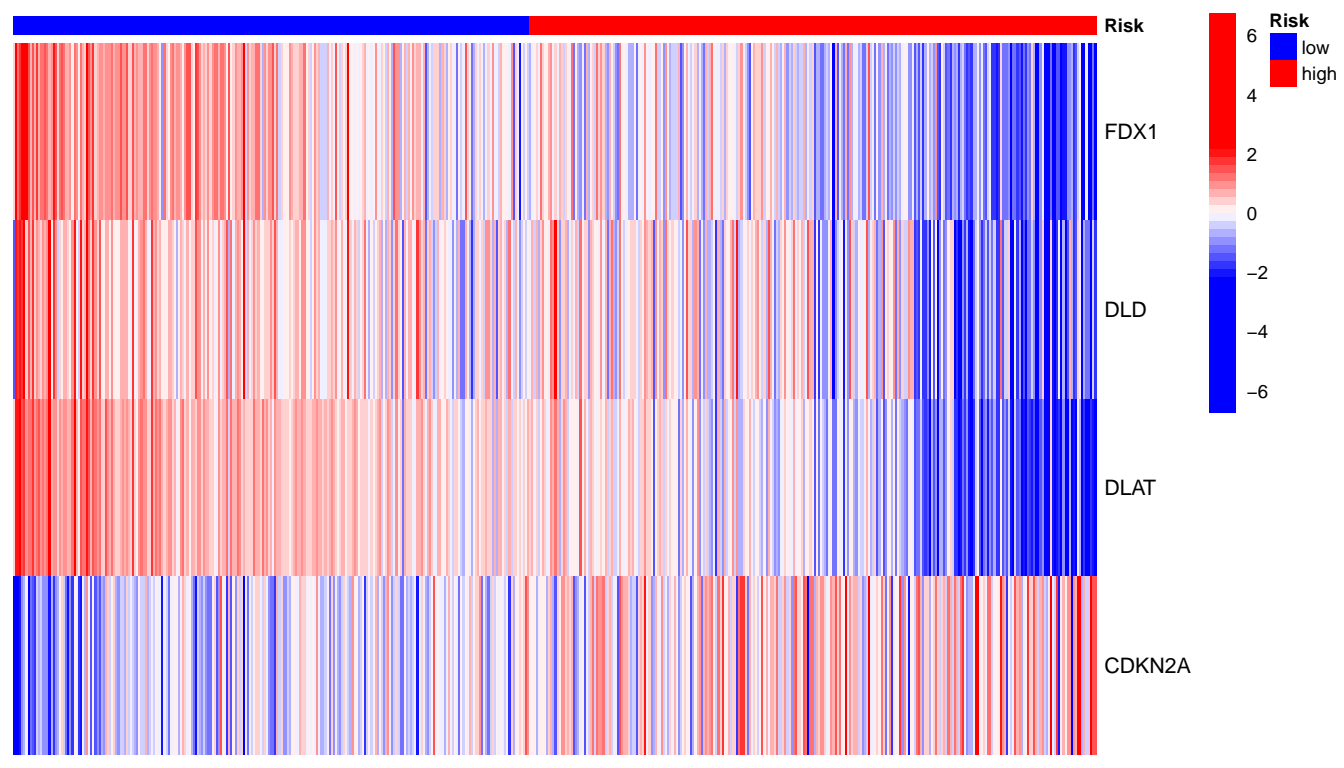

Supplement: Supplementary file 4 [file DataSheet1.ZIP › RAW.data/18/all.heatmap.pdf]

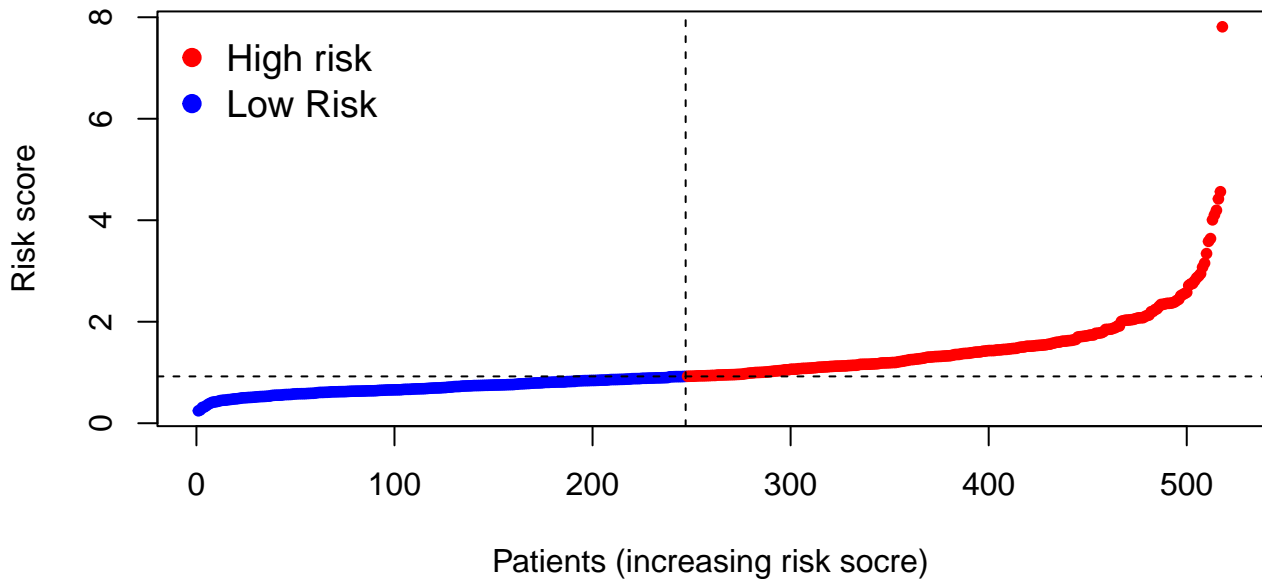

Supplement: Supplementary file 4 [file DataSheet1.ZIP › RAW.data/18/all.riskScore.pdf]

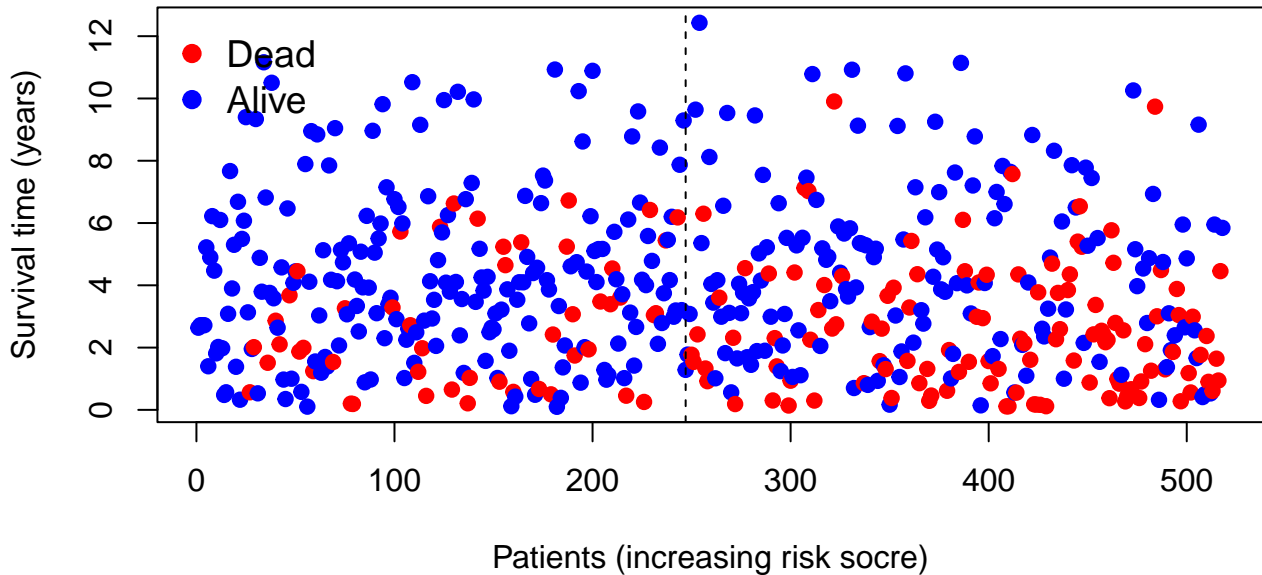

Supplement: Supplementary file 4 [file DataSheet1.ZIP › RAW.data/18/all.survStat.pdf]

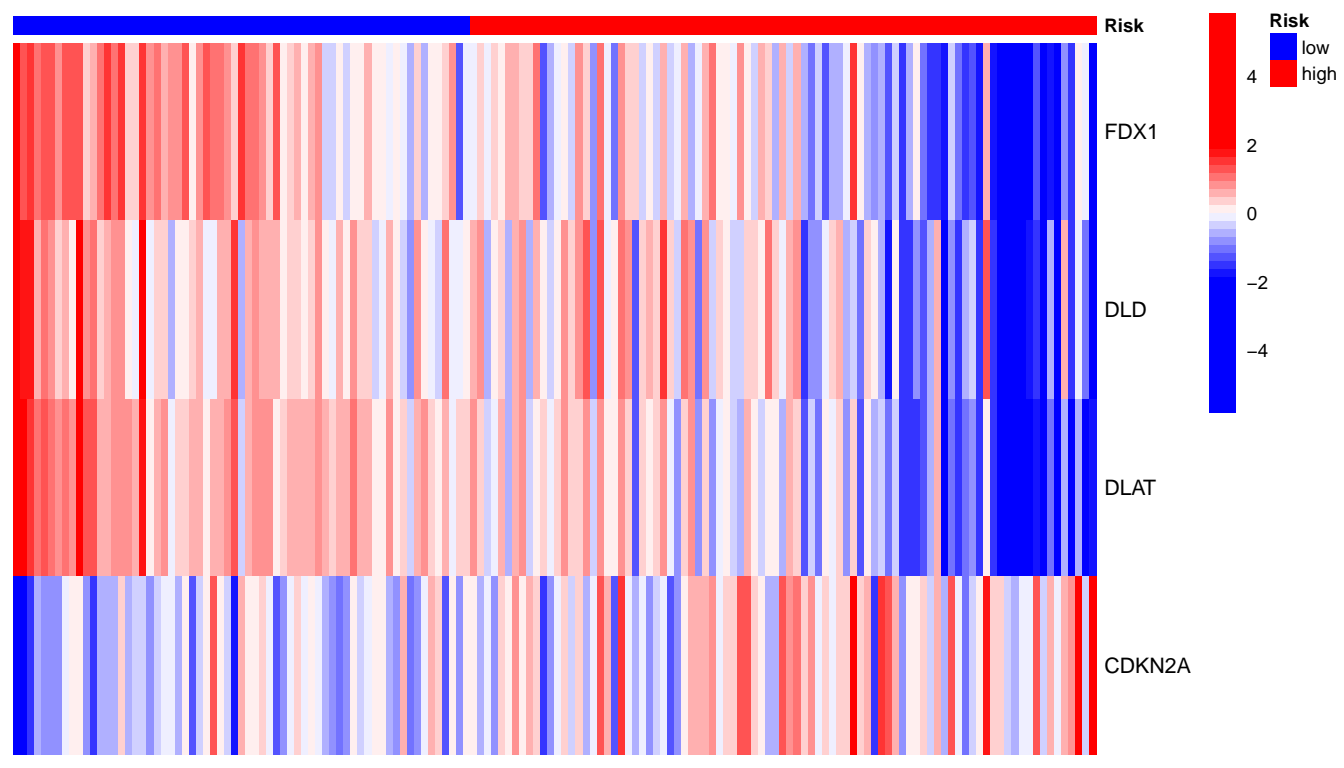

Supplement: Supplementary file 4 [file DataSheet1.ZIP › RAW.data/18/test.heatmap.pdf]

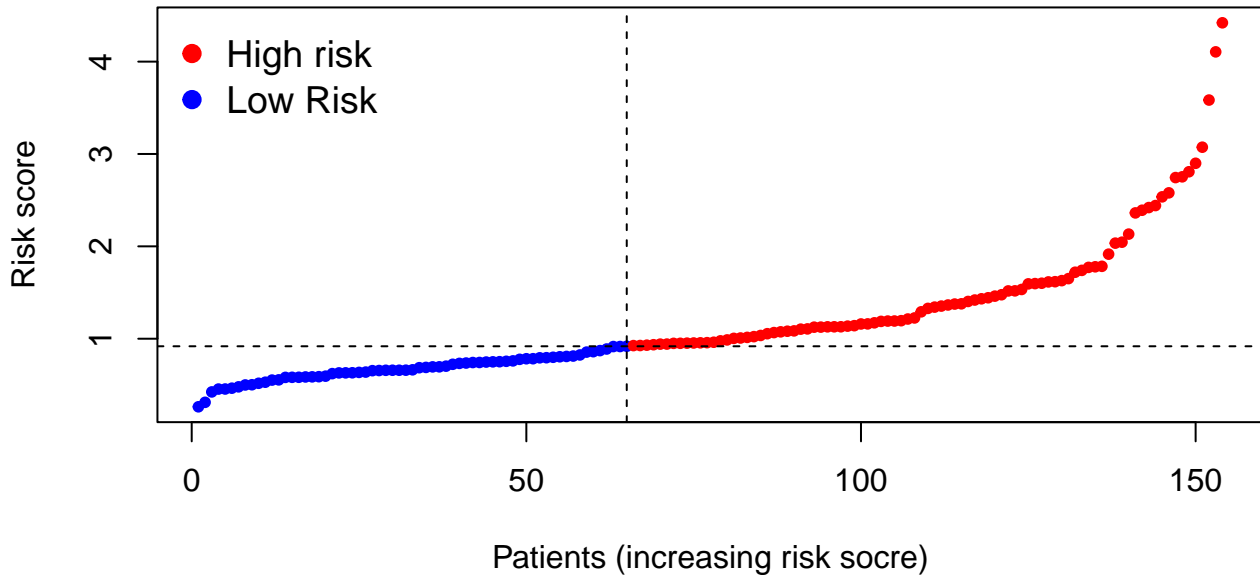

Supplement: Supplementary file 4 [file DataSheet1.ZIP › RAW.data/18/test.riskScore.pdf]

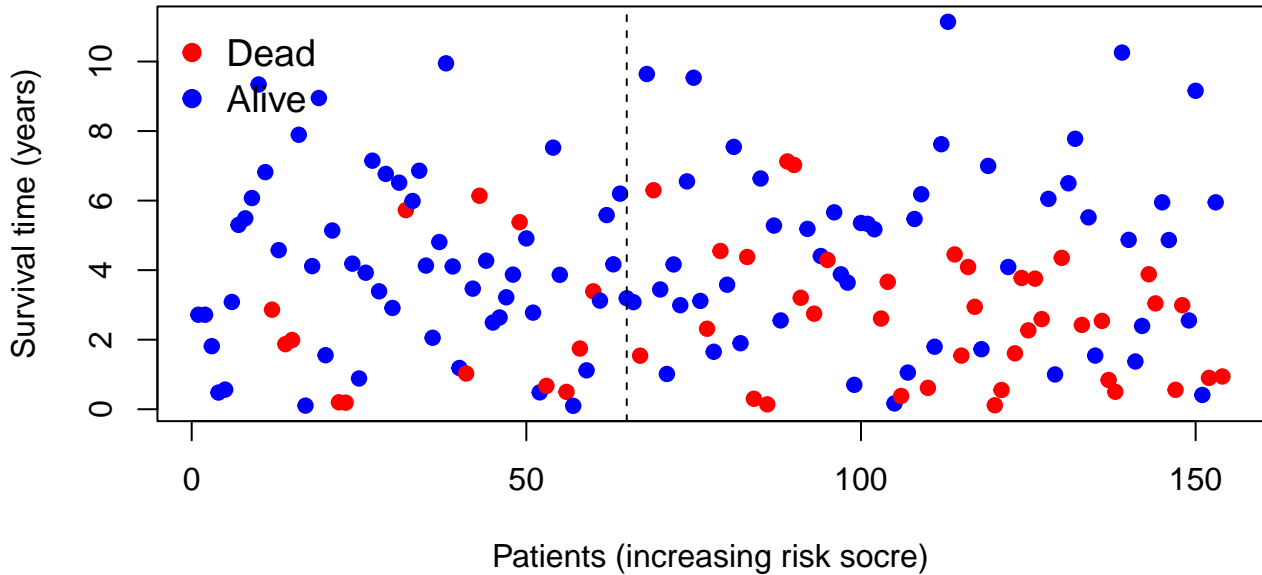

Supplement: Supplementary file 4 [file DataSheet1.ZIP › RAW.data/18/test.survStat.pdf]

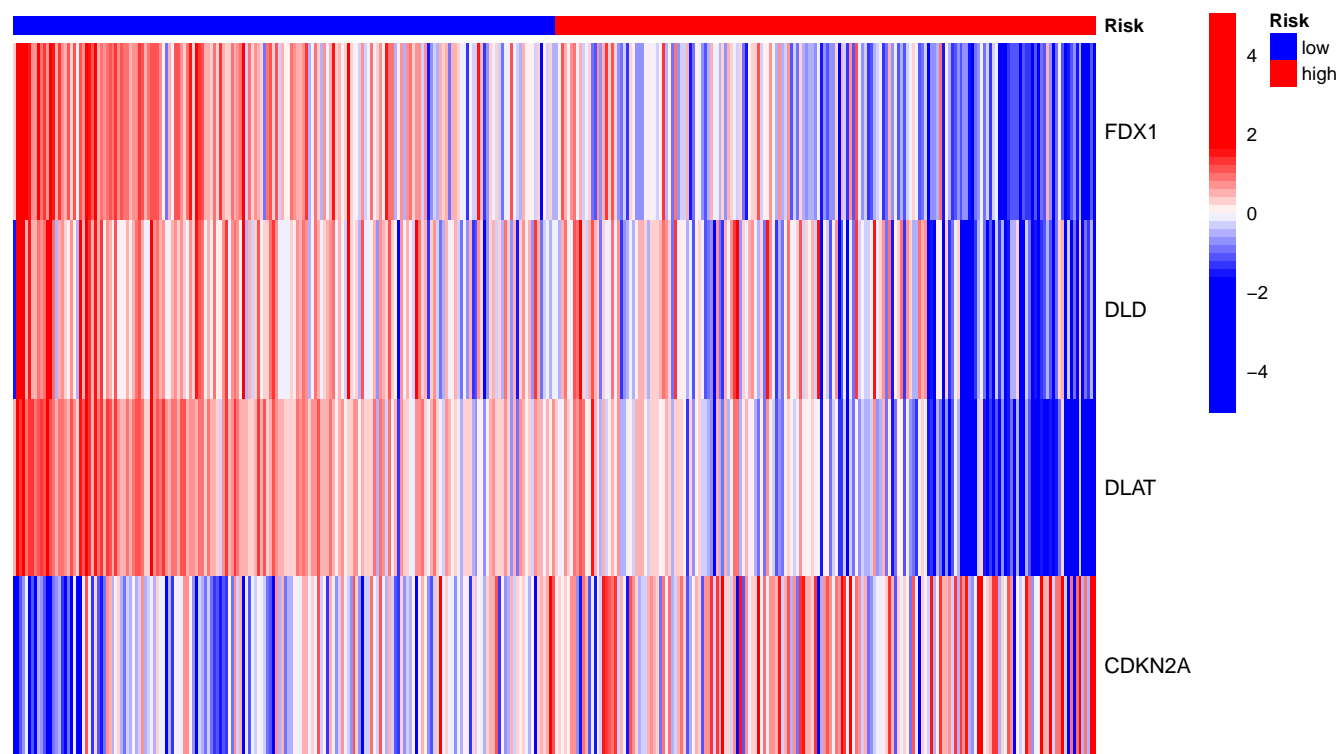

Supplement: Supplementary file 4 [file DataSheet1.ZIP › RAW.data/18/train.heatmap.pdf]

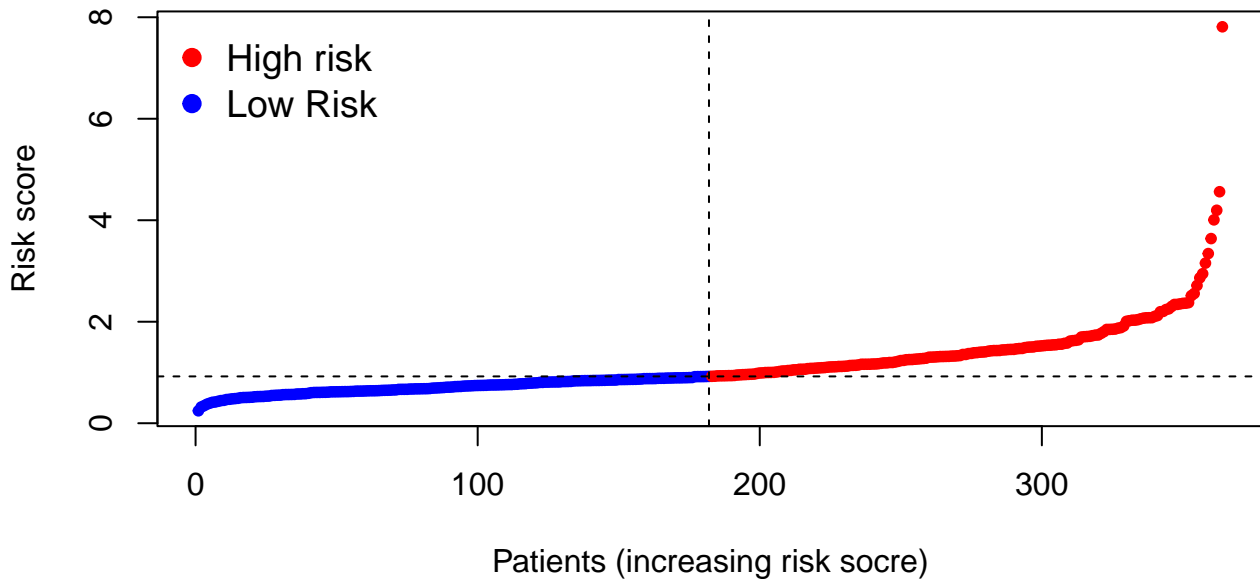

Supplement: Supplementary file 4 [file DataSheet1.ZIP › RAW.data/18/train.riskScore.pdf]

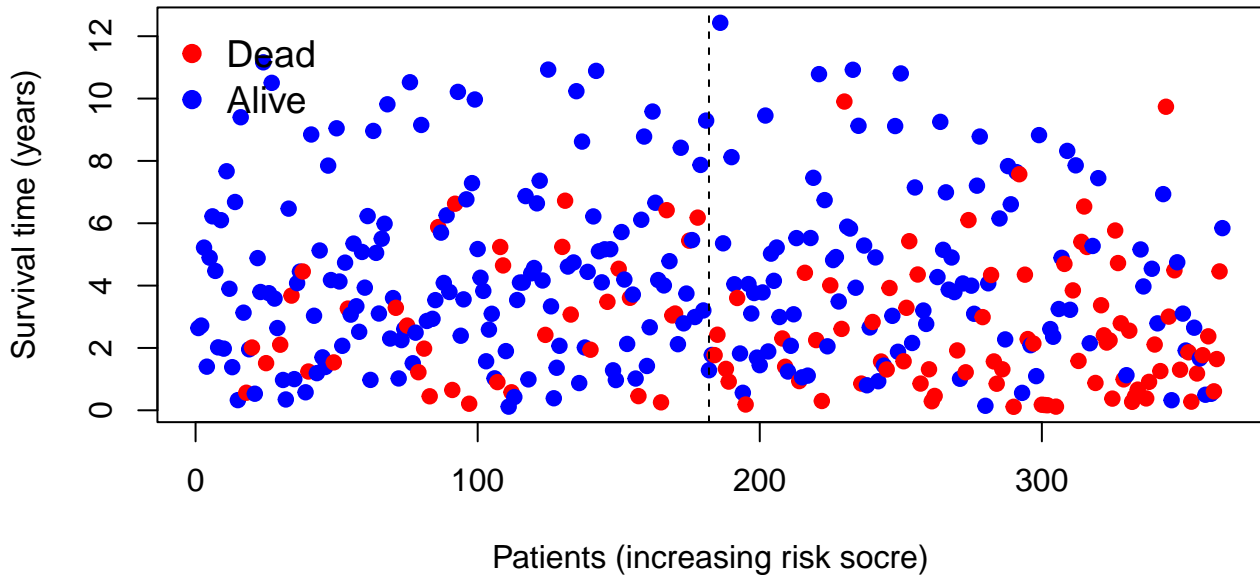

Supplement: Supplementary file 4 [file DataSheet1.ZIP › RAW.data/18/train.survStat.pdf]

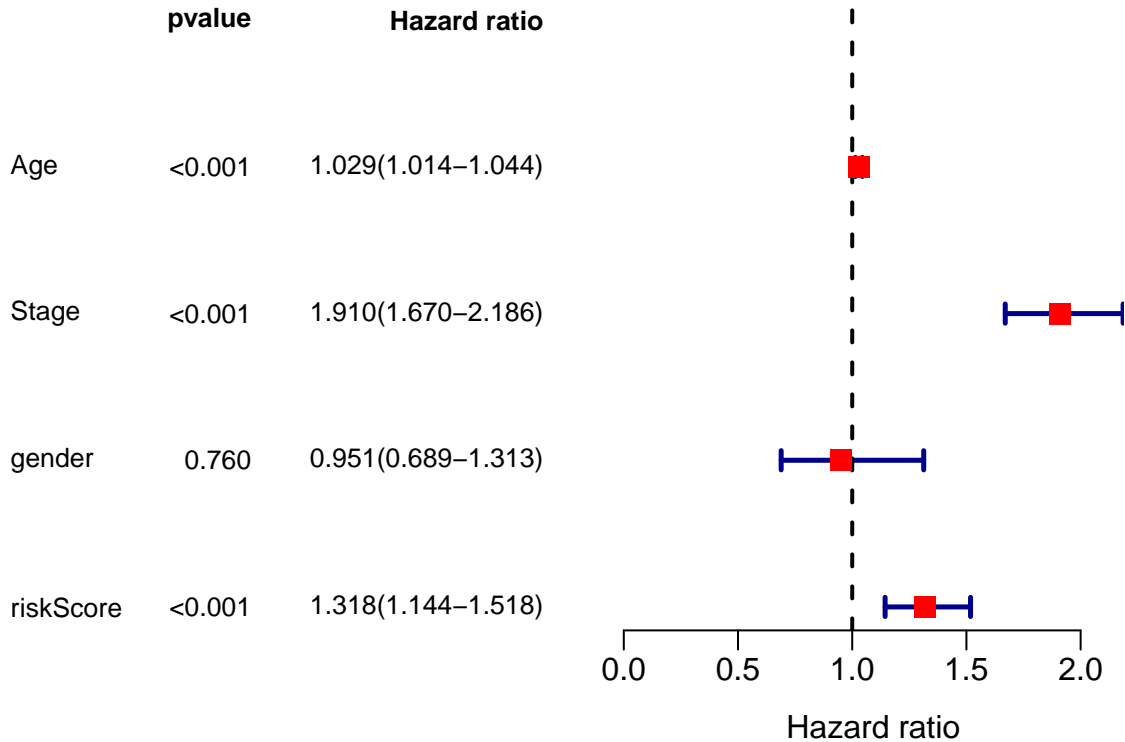

Supplement: Supplementary file 4 [file DataSheet1.ZIP › RAW.data/19/all.multiCox.pdf]

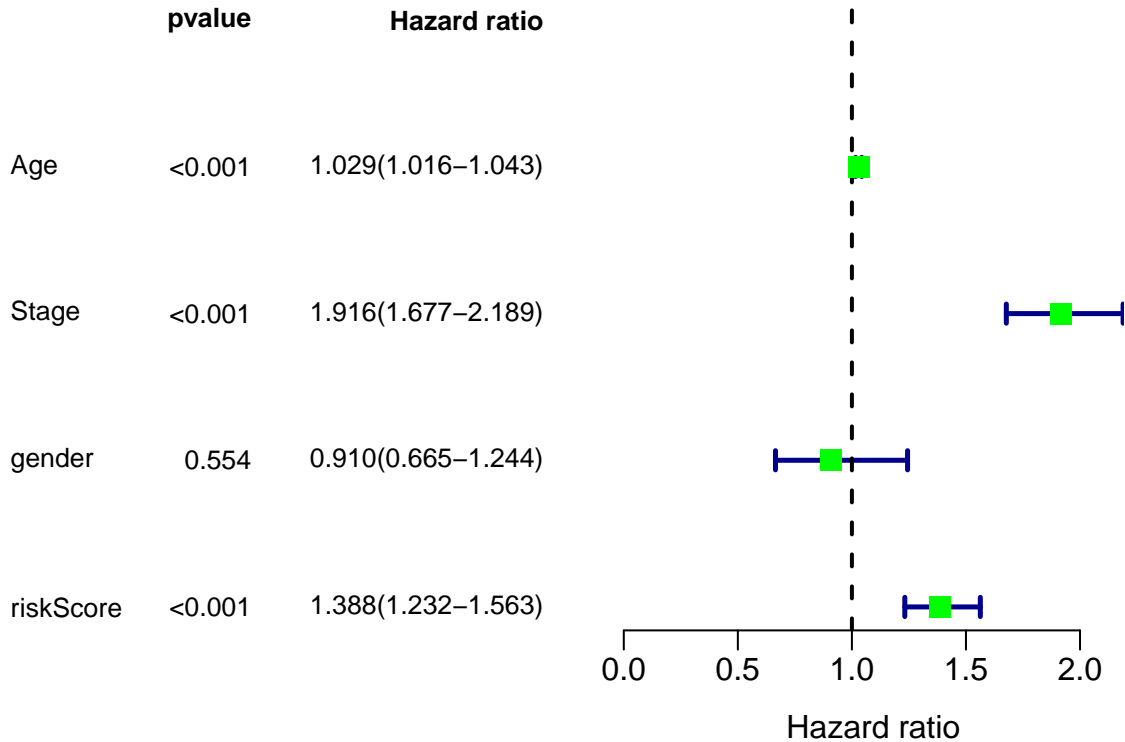

Supplement: Supplementary file 4 [file DataSheet1.ZIP › RAW.data/19/all.uniCox.pdf]

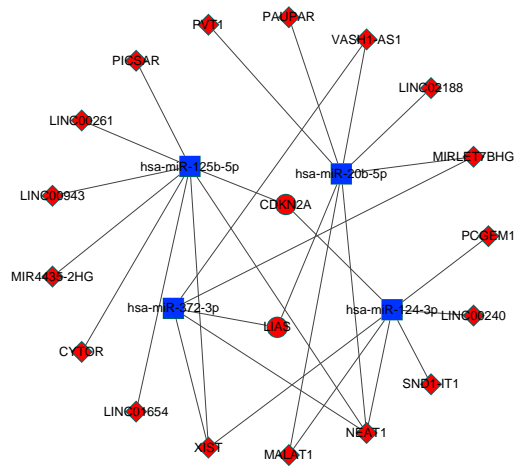

Supplement: Supplementary file 4 [file DataSheet1.ZIP › RAW.data/2.mutate/1ceRNA/n.txt.pdf]

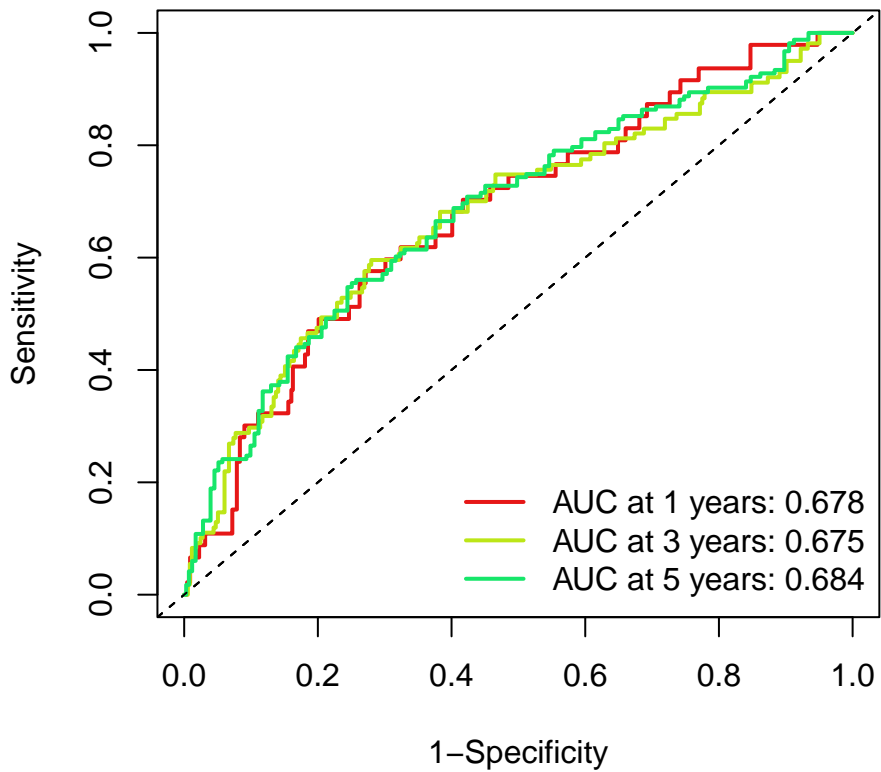

Supplement: Supplementary file 4 [file DataSheet1.ZIP › RAW.data/20/ROC.pdf]

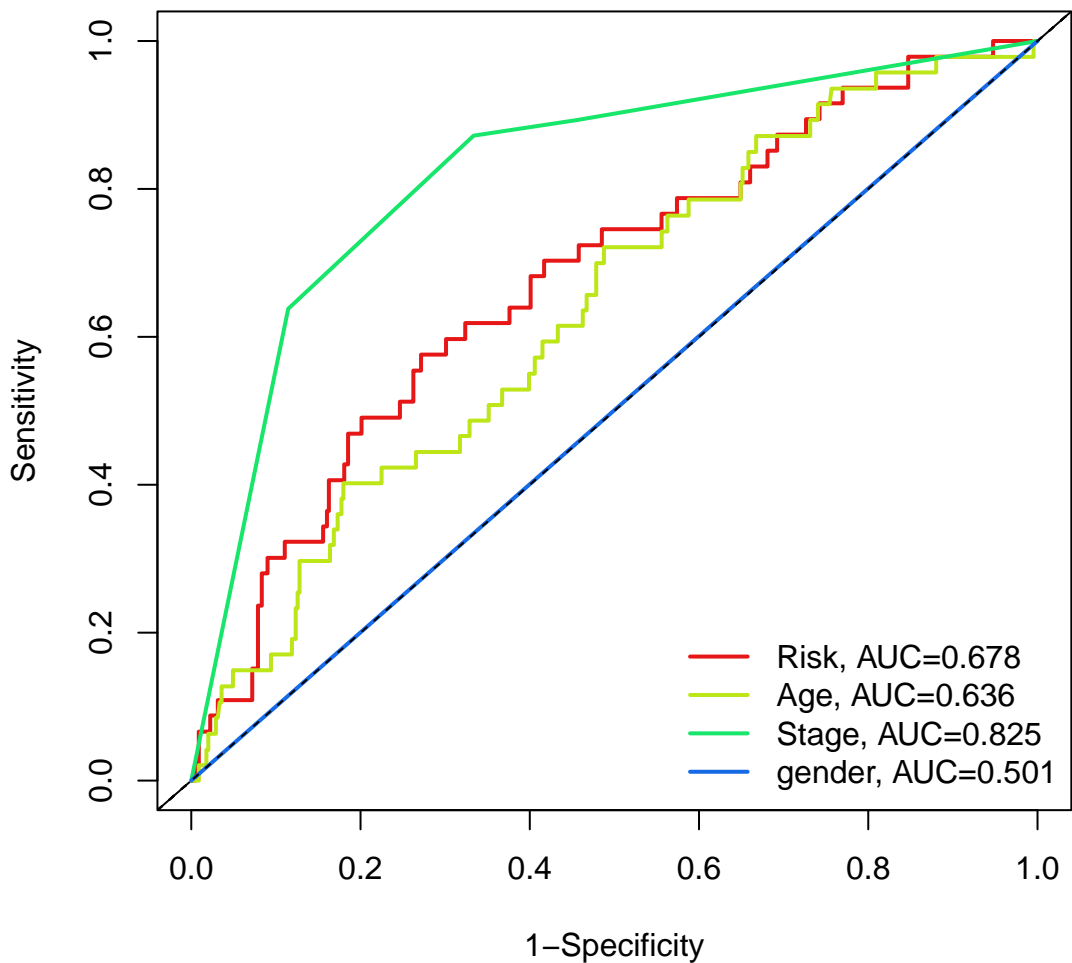

Supplement: Supplementary file 4 [file DataSheet1.ZIP › RAW.data/20/cliROC.pdf]

testing cohort

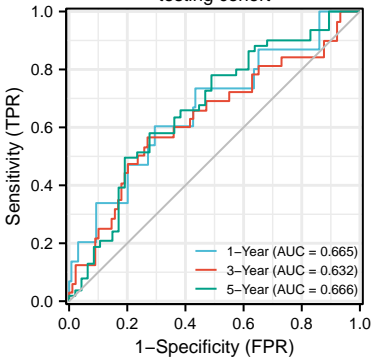

Supplement: Supplementary file 4 [file DataSheet1.ZIP › RAW.data/20/╩▒╝Σ╥└└╡ROCtest.pdf]

training cohort

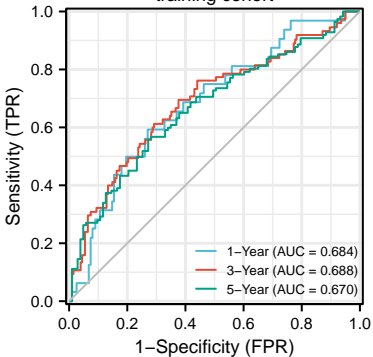

Supplement: Supplementary file 4 [file DataSheet1.ZIP › RAW.data/20/╩▒╝Σ╥└└╡ROCtraining.pdf]

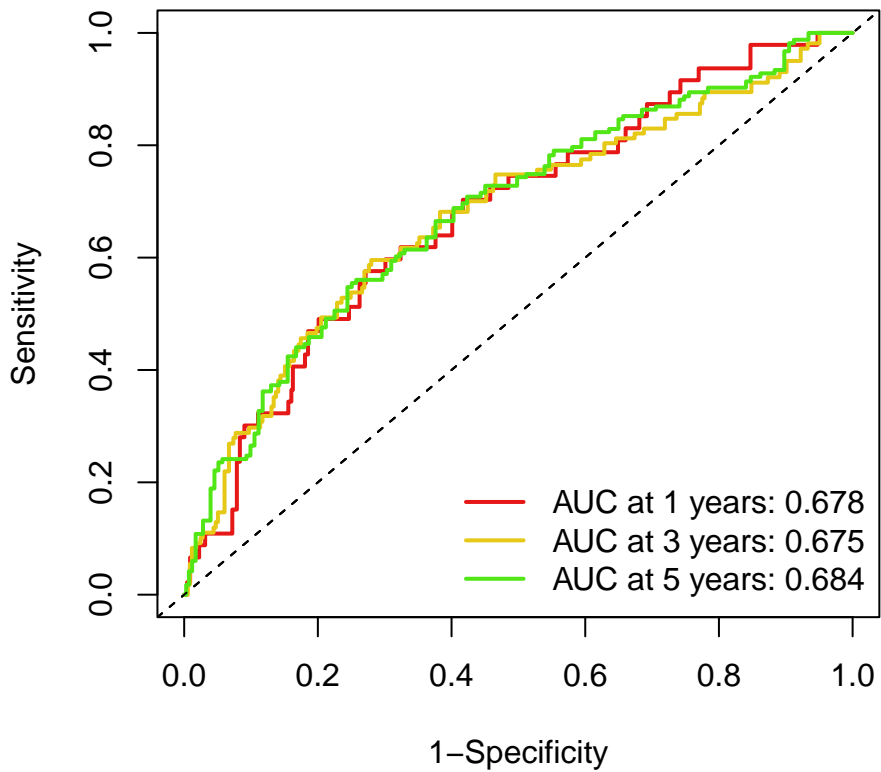

Supplement: Supplementary file 4 [file DataSheet1.ZIP › RAW.data/20TNM/ROC.pdf]

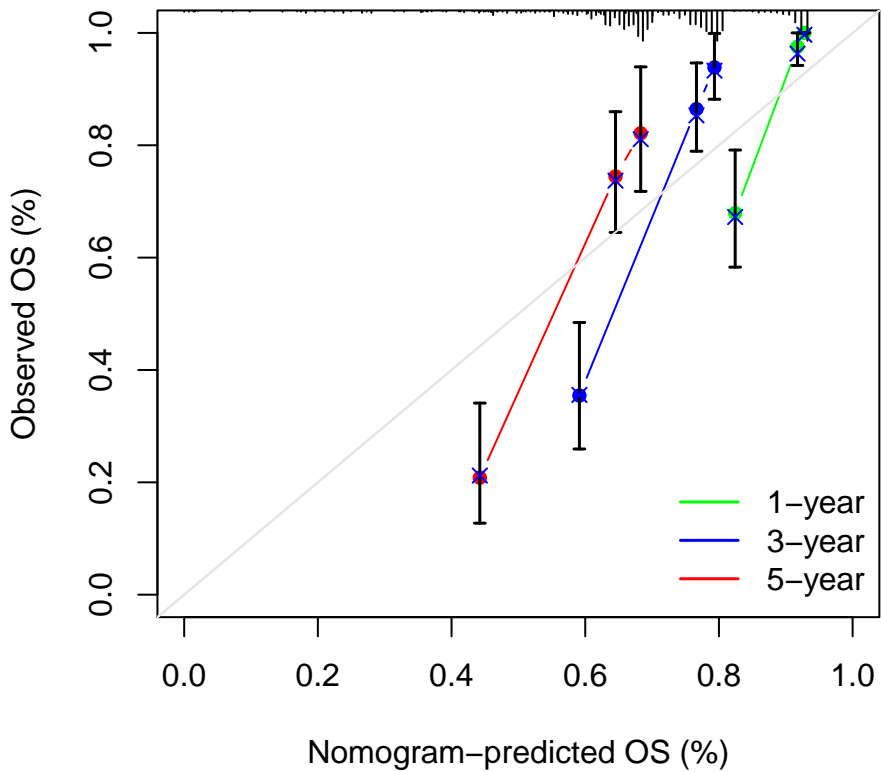

Supplement: Supplementary file 4 [file DataSheet1.ZIP › RAW.data/22/calibration.pdf]

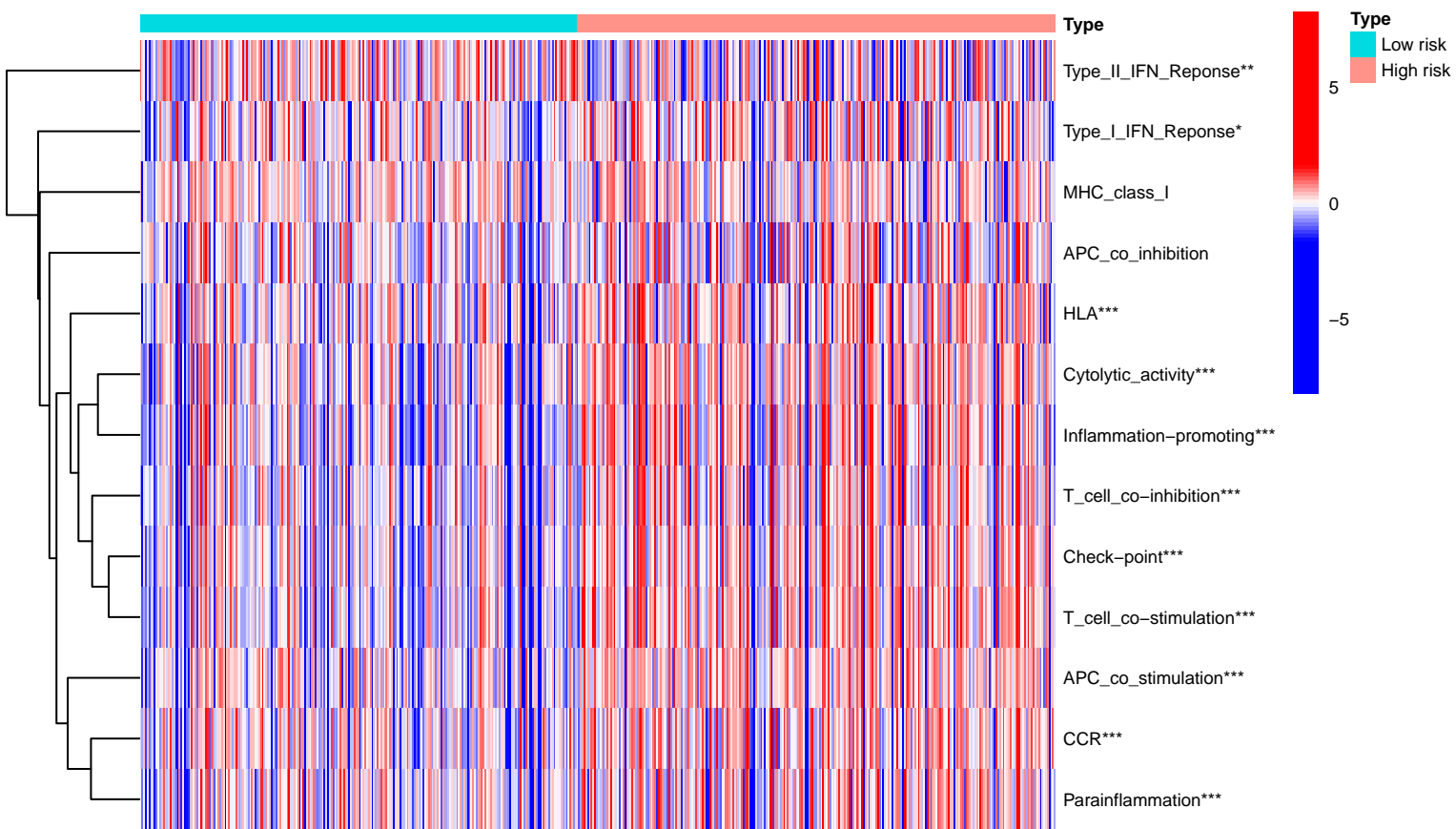

Supplement: Supplementary file 4 [file DataSheet1.ZIP › RAW.data/28.ssgsea/heatmap.pdf]

Risk    ■ Low-risk    ■ High-risk

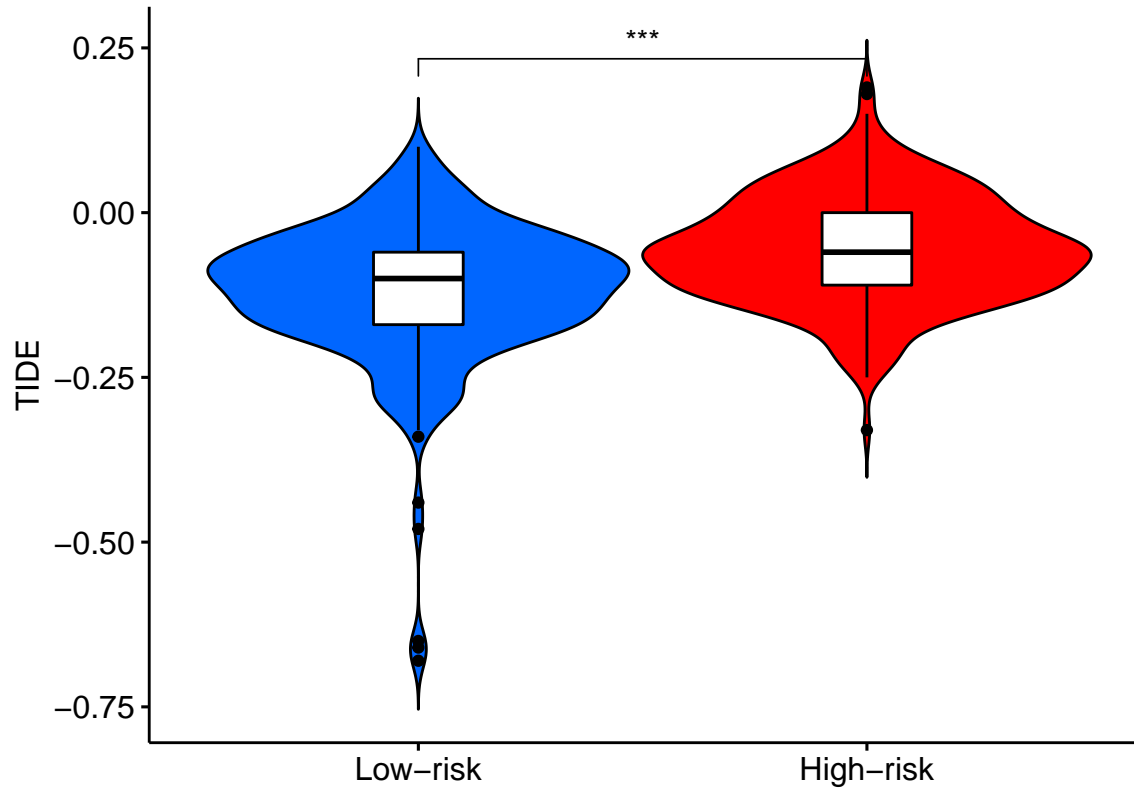

Supplement: Supplementary file 4 [file DataSheet1.ZIP › RAW.data/32/TIDE.pdf]

Risk 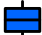 low 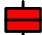 high

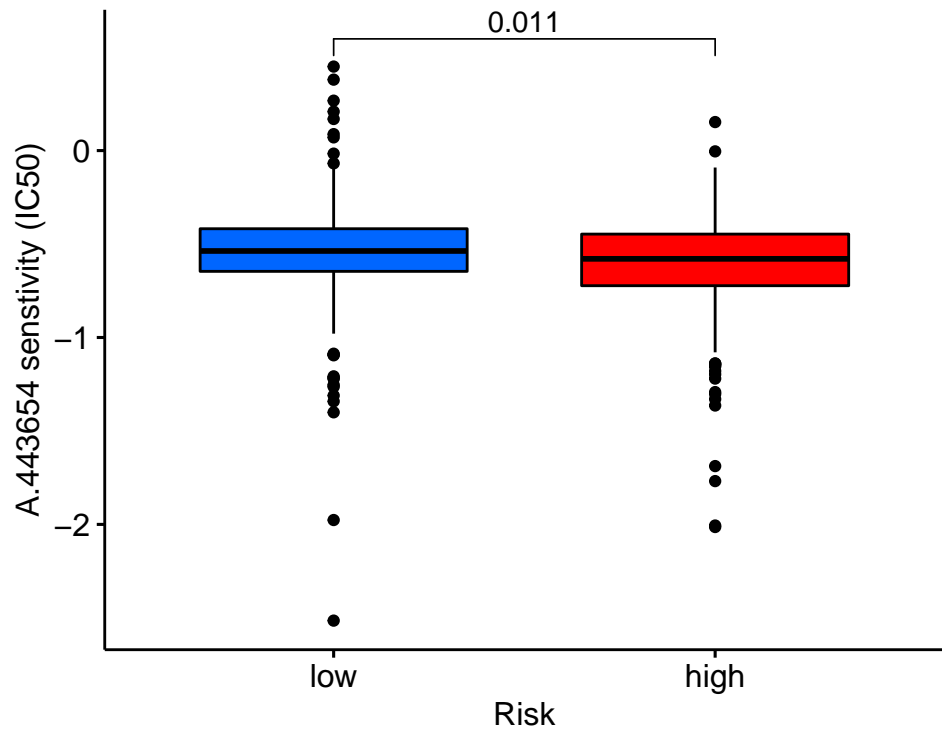

Supplement: Supplementary file 4 [file DataSheet1.ZIP › RAW.data/33/durgSenstivity.A.443654.pdf]

Risk 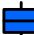 low 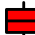 high

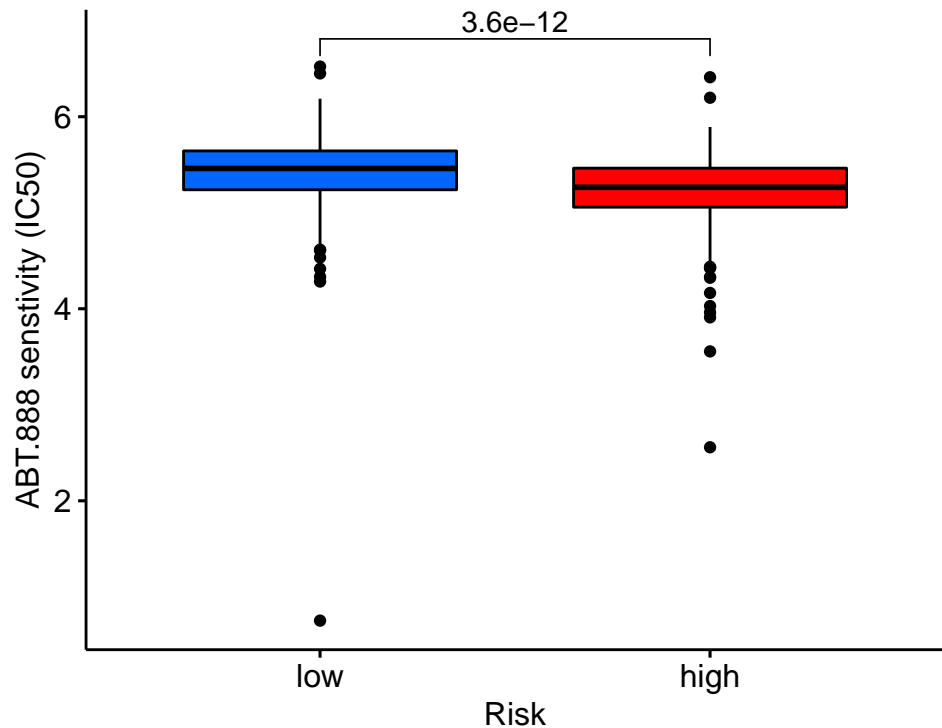

Supplement: Supplementary file 4 [file DataSheet1.ZIP › RAW.data/33/durgSenstivity.ABT.888.pdf]

Risk 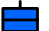 low 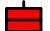 high

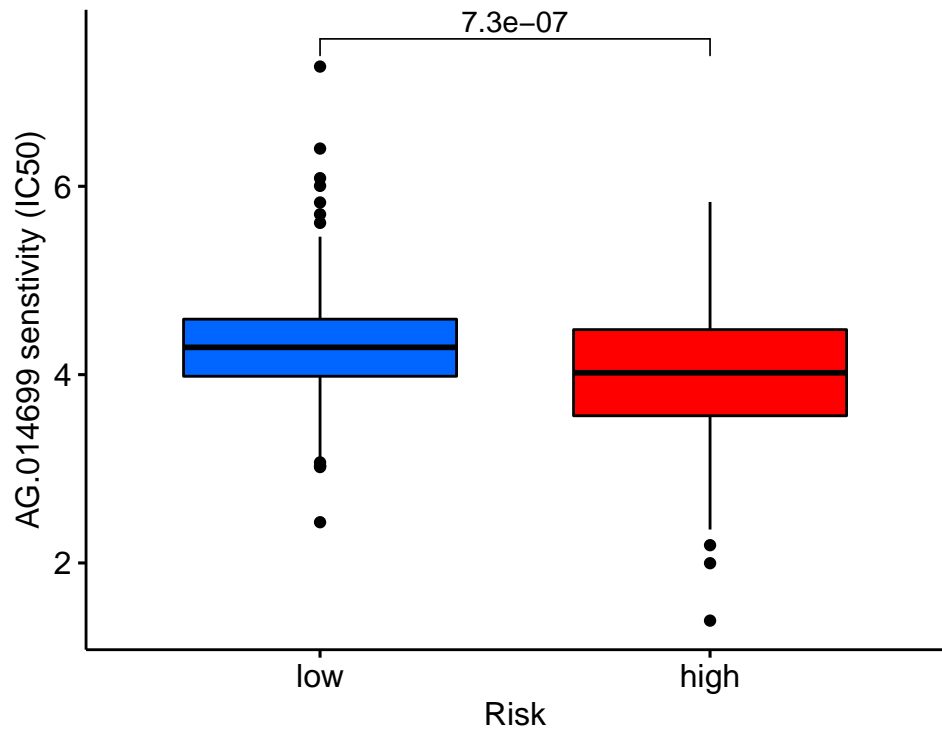

Supplement: Supplementary file 4 [file DataSheet1.ZIP › RAW.data/33/durgSenstivity.AG.014699.pdf]

Risk 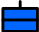 low 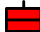 high

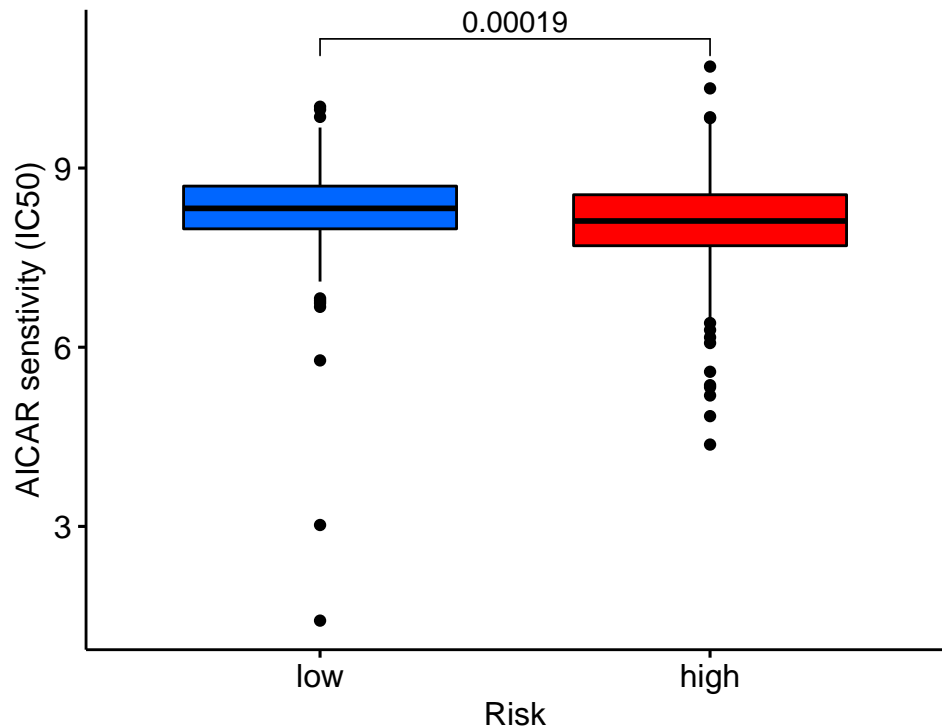

Supplement: Supplementary file 4 [file DataSheet1.ZIP › RAW.data/33/durgSenstivity.AICAR.pdf]

Risk 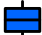 low 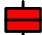 high

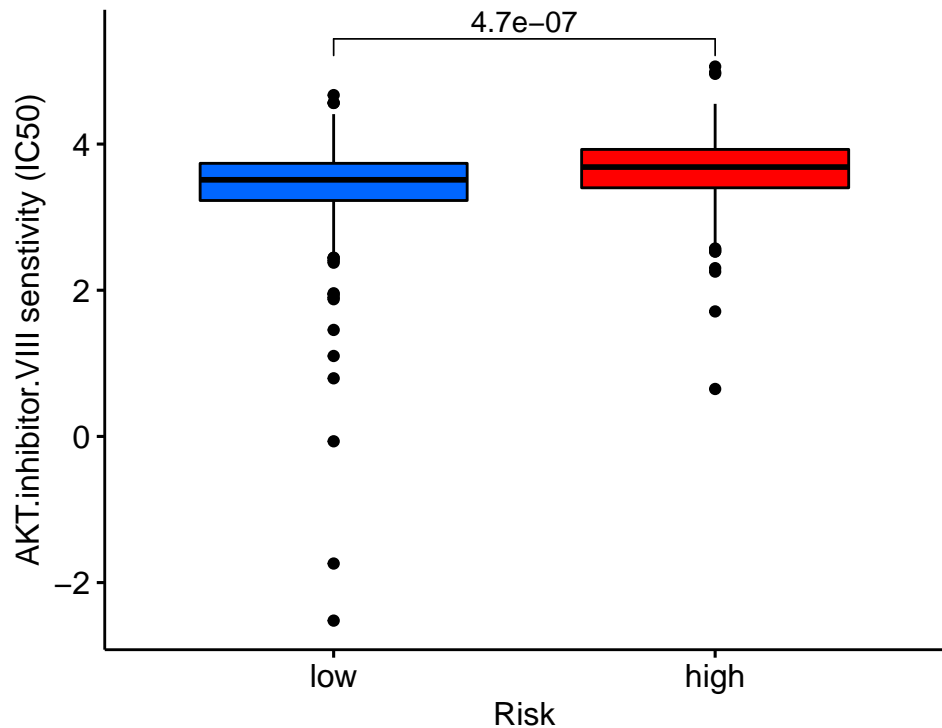

Supplement: Supplementary file 4 [file DataSheet1.ZIP › RAW.data/33/durgSenstivity.AKT.inhibitor.VIII.pdf]

Risk 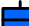 low 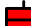 high

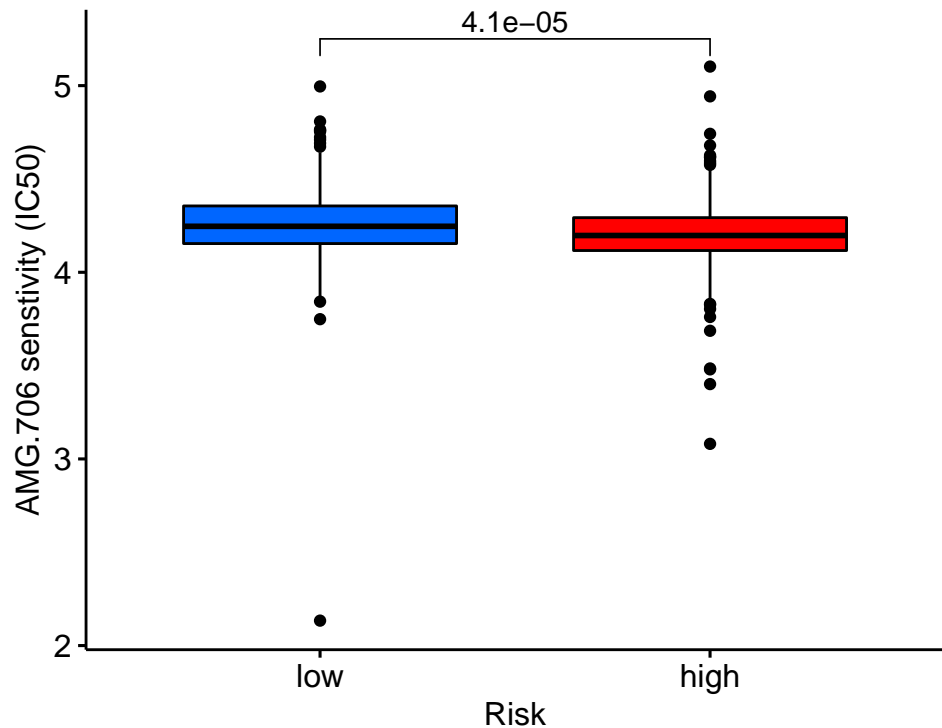

Supplement: Supplementary file 4 [file DataSheet1.ZIP › RAW.data/33/durgSenstivity.AMG.706.pdf]

Risk 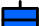 low 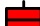 high

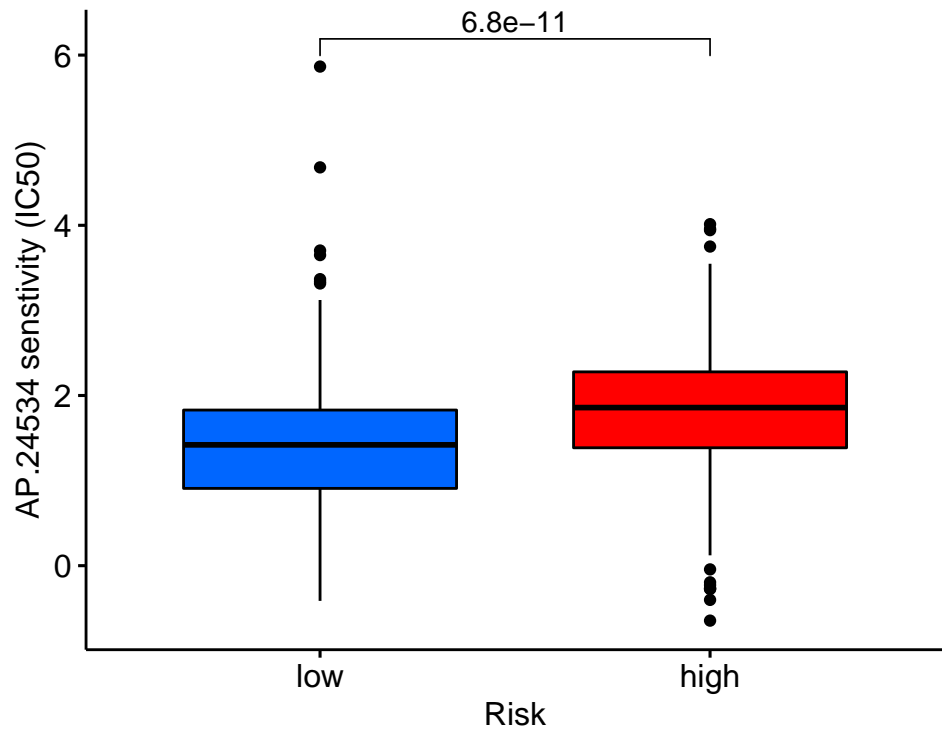

Supplement: Supplementary file 4 [file DataSheet1.ZIP › RAW.data/33/durgSenstivity.AP.24534.pdf]

Risk low high

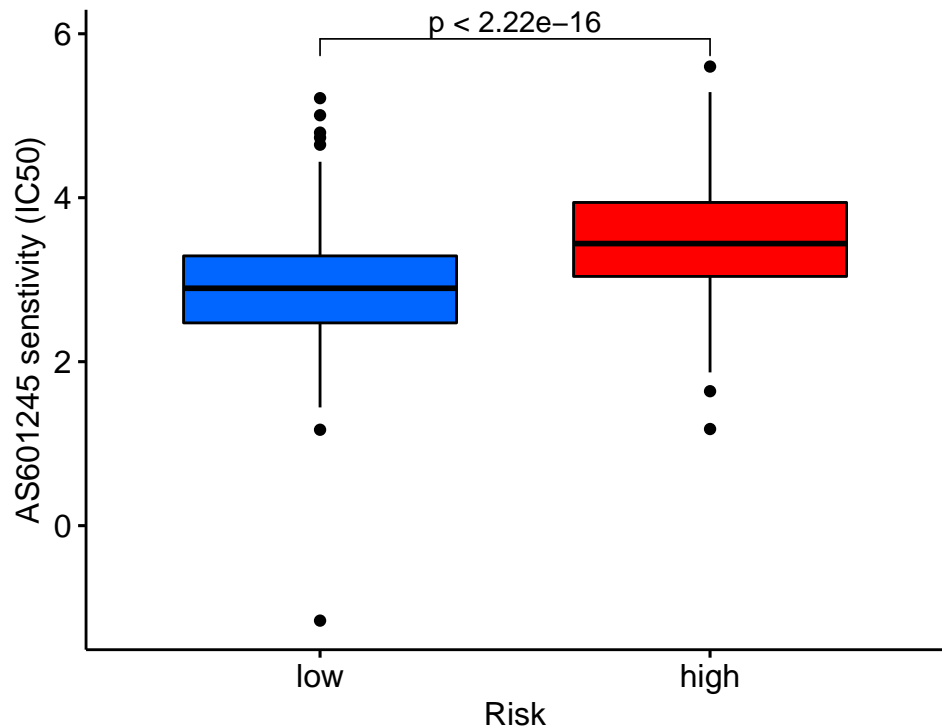

Supplement: Supplementary file 4 [file DataSheet1.ZIP › RAW.data/33/durgSenstivity.AS601245.pdf]

Risk 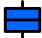 low 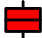 high

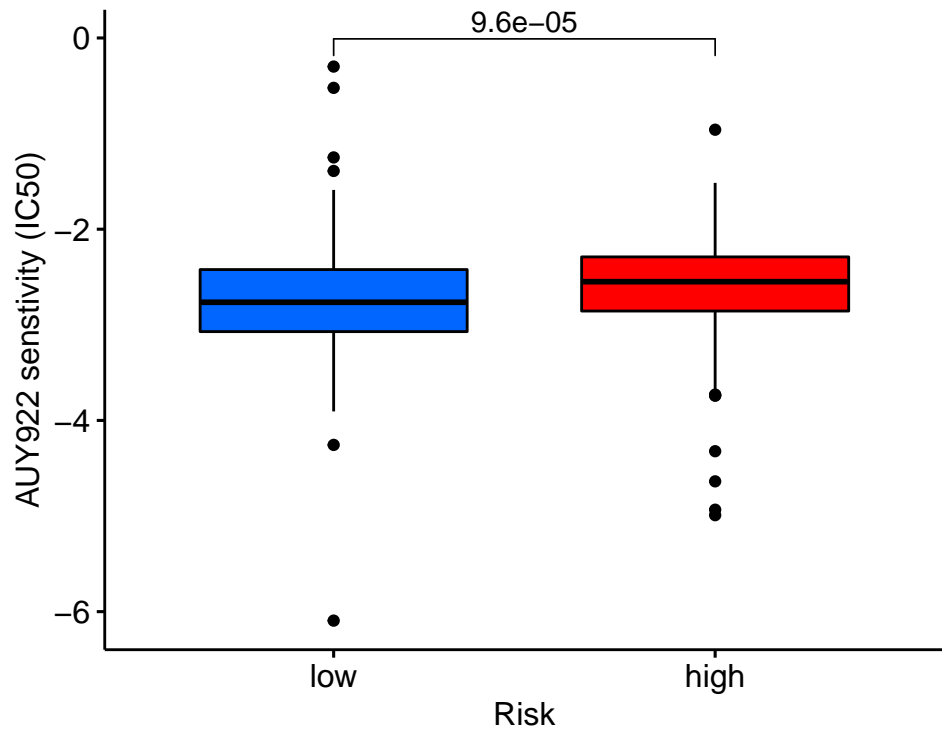

Supplement: Supplementary file 4 [file DataSheet1.ZIP › RAW.data/33/durgSenstivity.AUY922.pdf]

Risk 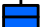 low 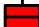 high

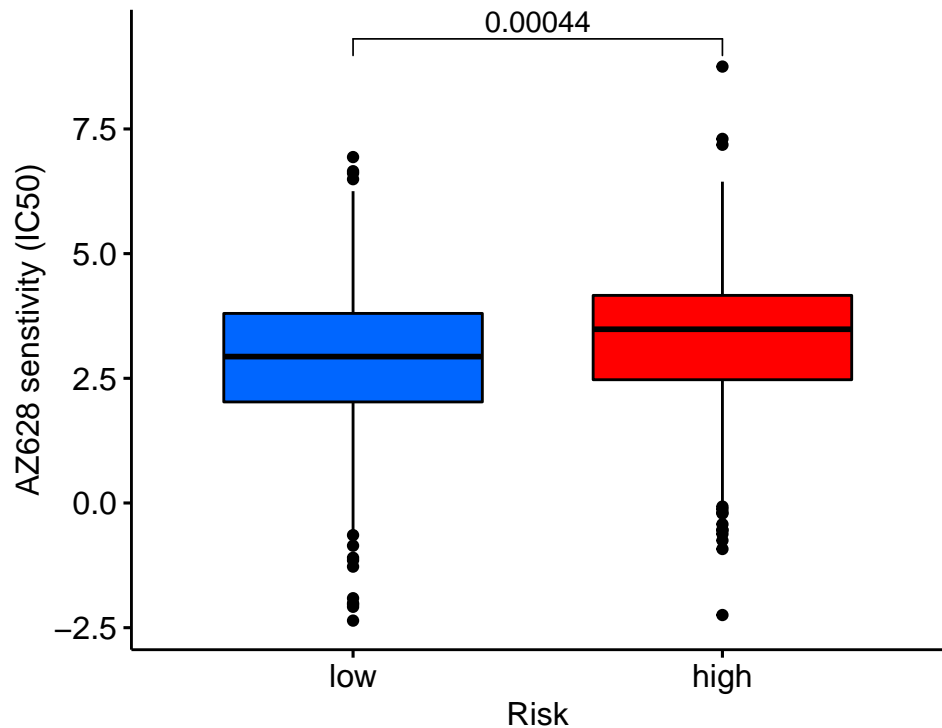

Supplement: Supplementary file 4 [file DataSheet1.ZIP › RAW.data/33/durgSenstivity.AZ628.pdf]

Risk 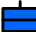 low 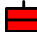 high

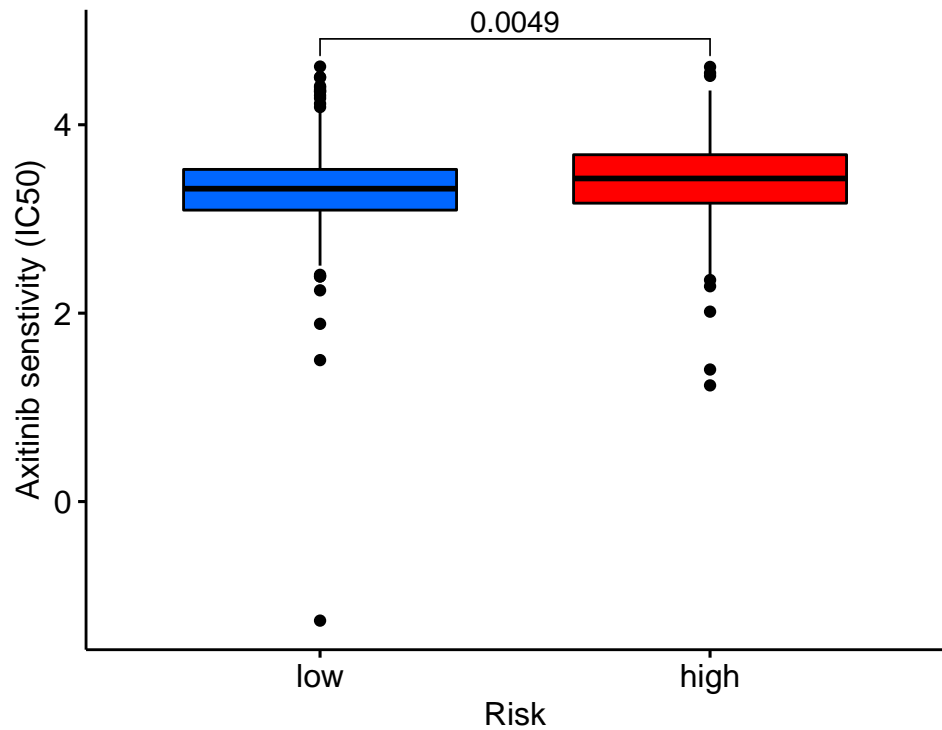

Supplement: Supplementary file 4 [file DataSheet1.ZIP › RAW.data/33/durgSenstivity.Axitinib.pdf]

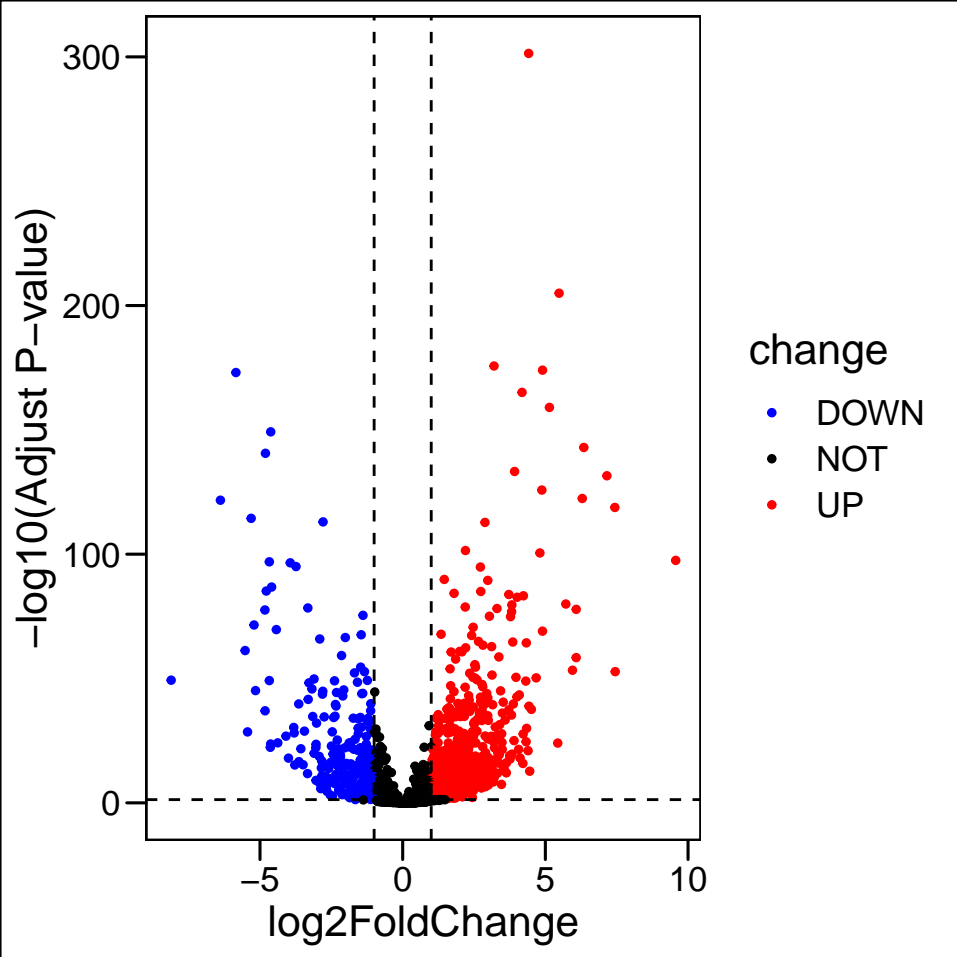

Supplement: Supplementary file 4 [file DataSheet1.ZIP › RAW.data/34ceRNA/lncRNA╗≡╔╜═╝.pdf]

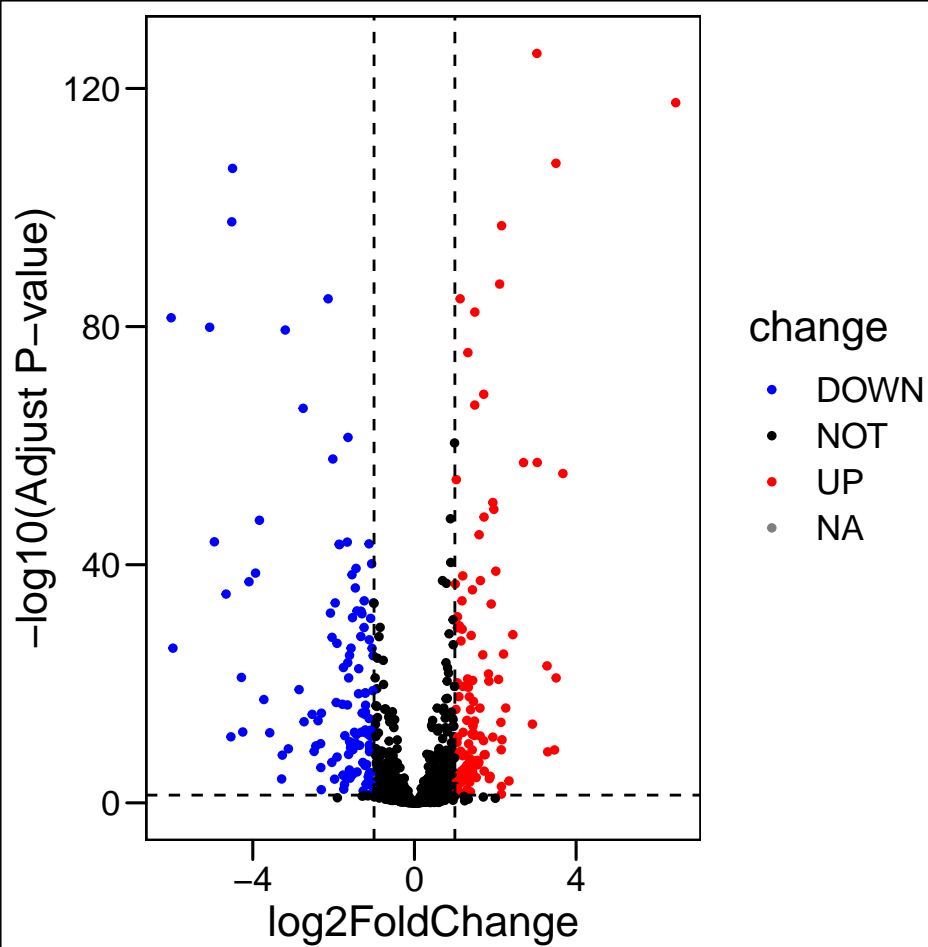

Supplement: Supplementary file 4 [file DataSheet1.ZIP › RAW.data/34ceRNA/miRNA╗≡╔╜.pdf]

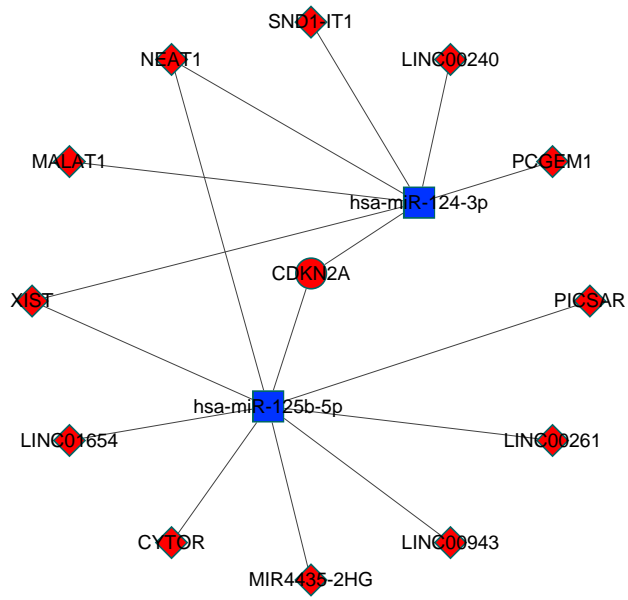

Supplement: Supplementary file 4 [file DataSheet1.ZIP › RAW.data/34ceRNA/n1.txt.pdf]

DElncRNAs

Copper death-related lncRNAs

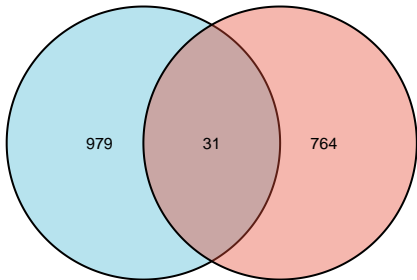

Supplement: Supplementary file 4 [file DataSheet1.ZIP › RAW.data/34ceRNA/╬1⁄4╢≈═╝lncRNA.pdf]

DEmiRNAs

Copper death-related miRNAs

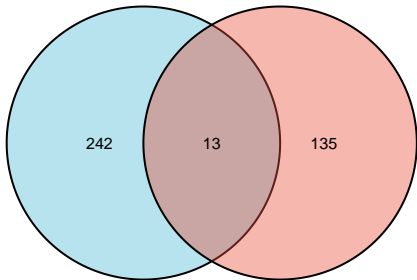

Supplement: Supplementary file 4 [file DataSheet1.ZIP › RAW.data/34ceRNA/╬1⁄4╢≈═╝miRNA.pdf]

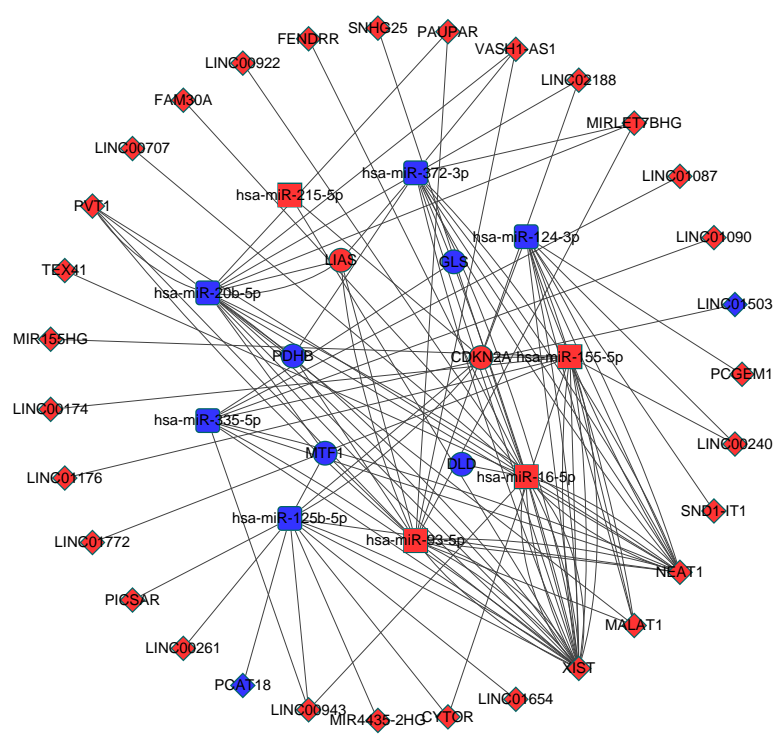

Supplement: Supplementary file 4 [file DataSheet1.ZIP › RAW.data/4/1.pdf]

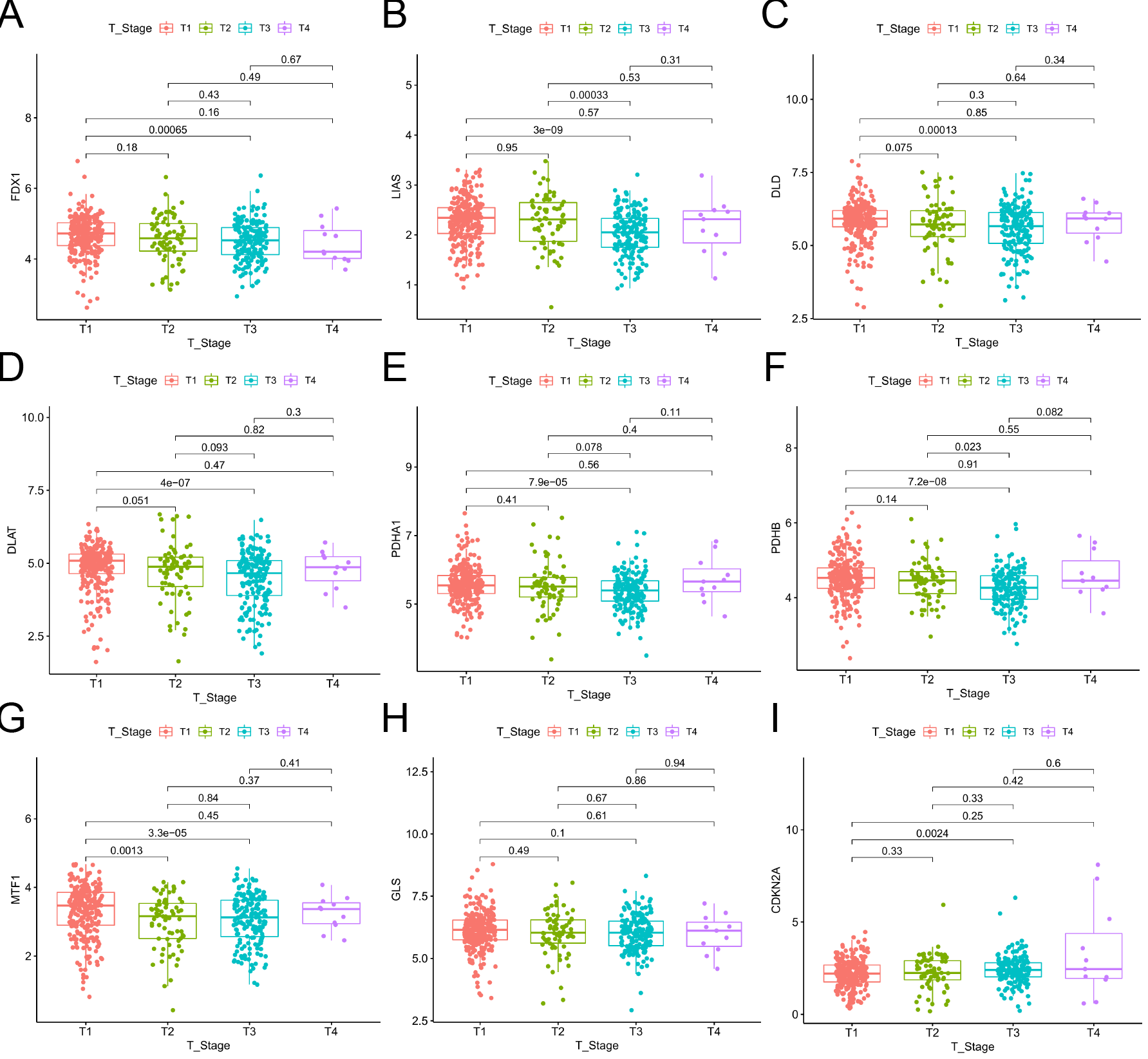

Supplement: Supplementary file 5 [file Image1.TIF]

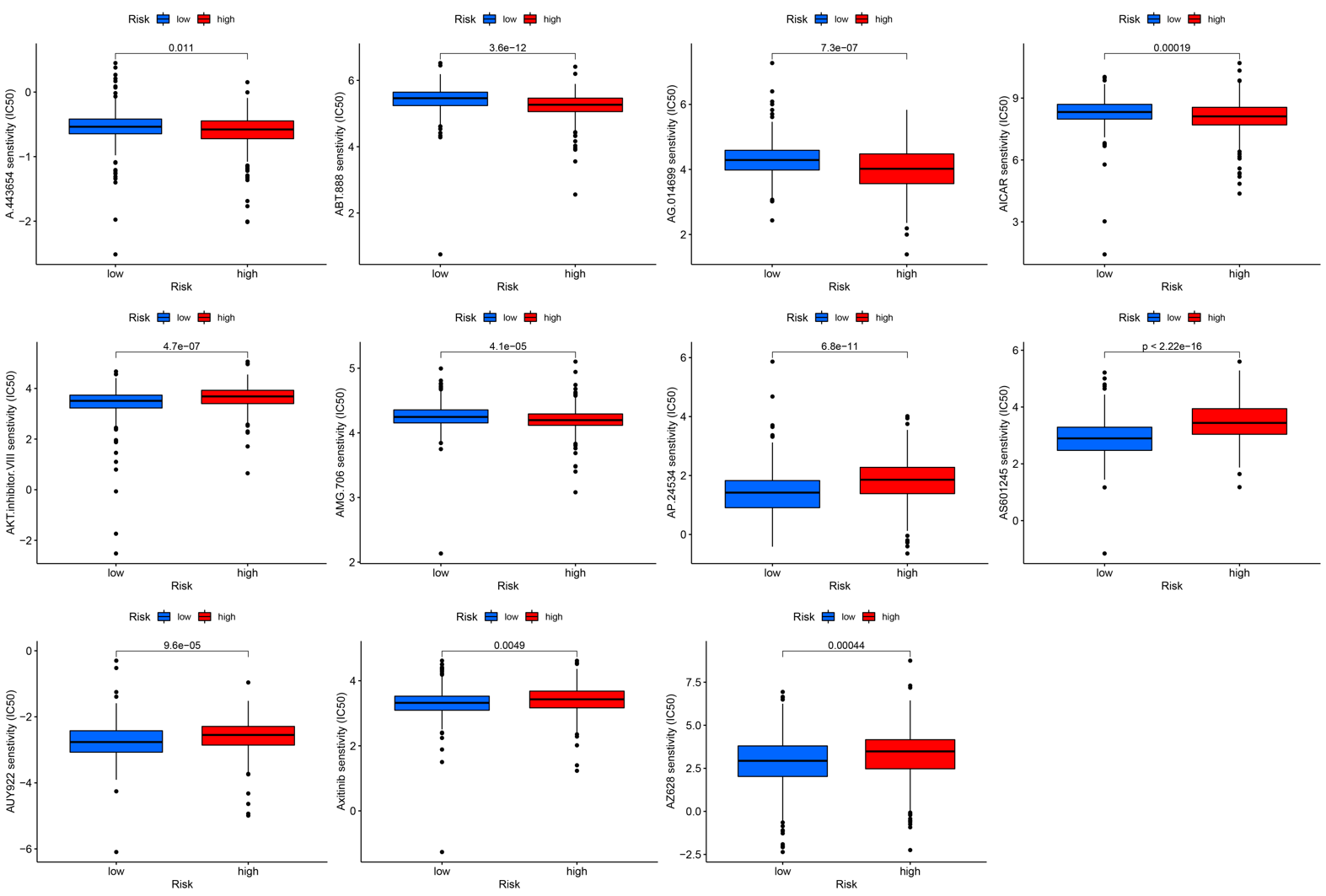

Supplement: Supplementary file 6 [file Image7.TIF]

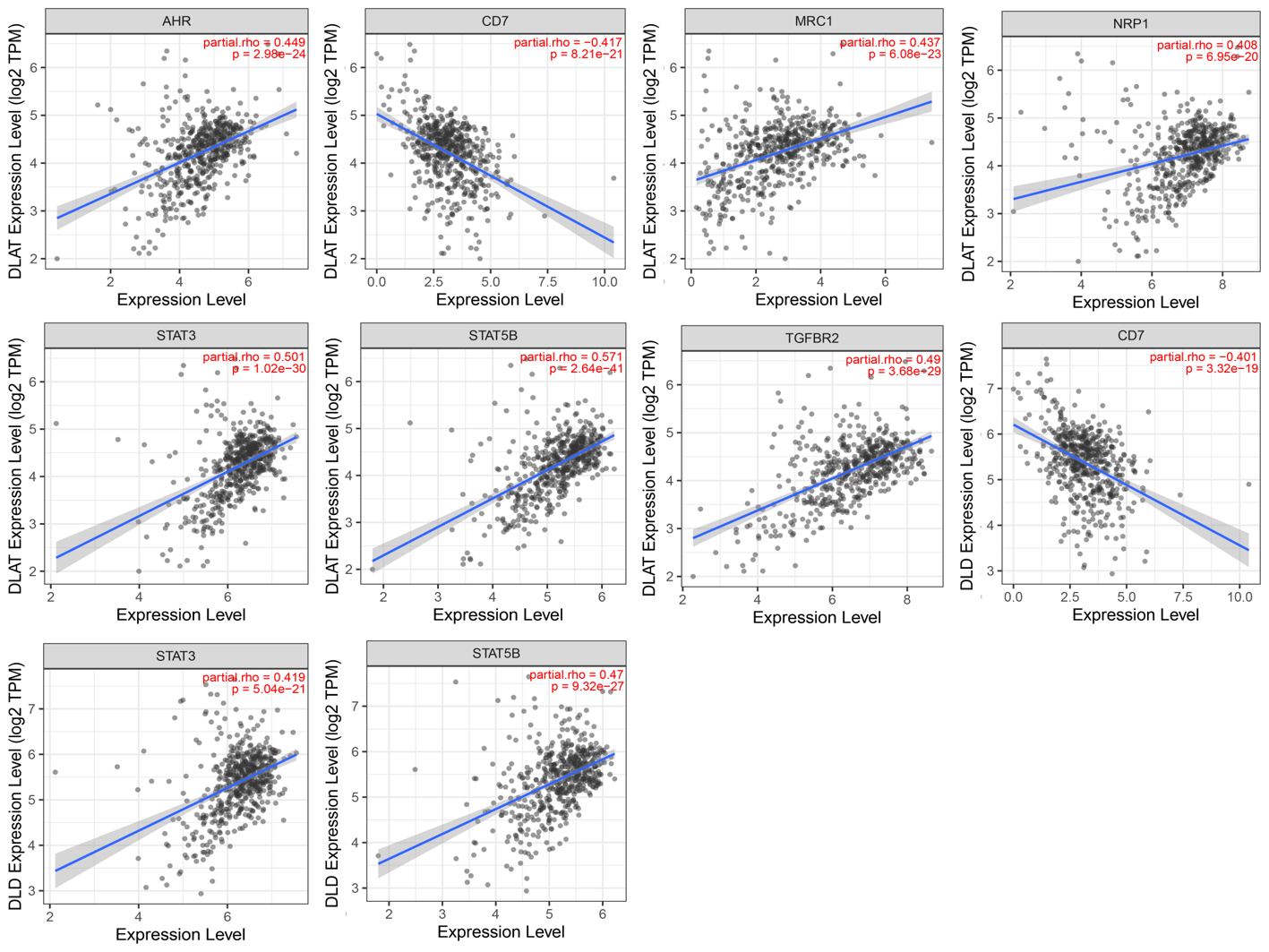

Supplement: Supplementary file 8 [file Image5.TIF]

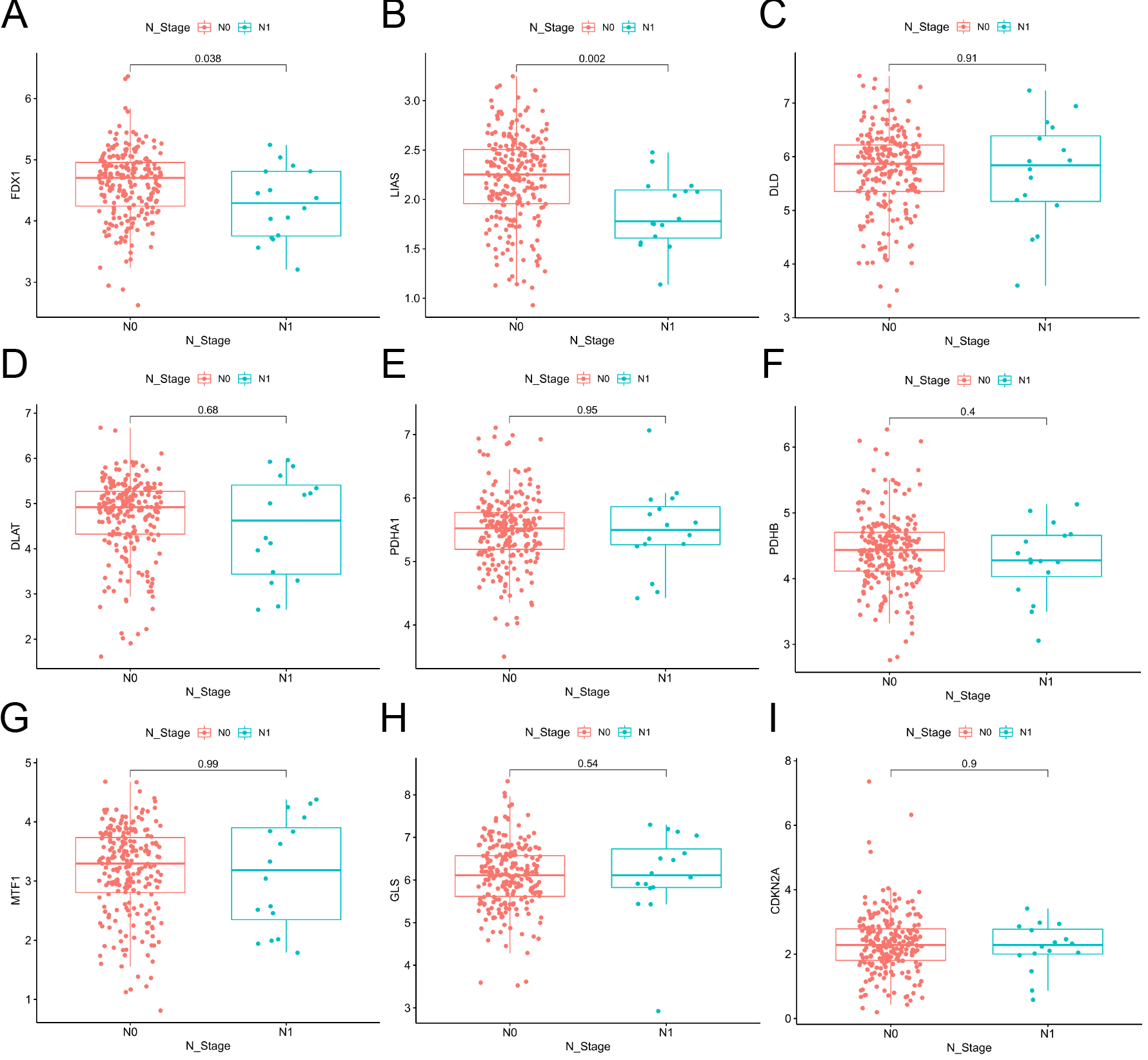

Supplement: Supplementary file 9 [file Image2.TIFF]
